# Identifying cancer-associated leukocyte profiles using high-resolution flow cytometry screening and machine learning

**DOI:** 10.3389/fimmu.2023.1211064

**Published:** 2023-08-03

**Authors:** David A. Simon Davis, Melissa Ritchie, Dillon Hammill, Jessica Garrett, Robert O. Slater, Naomi Otoo, Anna Orlov, Katharine Gosling, Jason Price, Desmond Yip, Kylie Jung, Farhan M. Syed, Ines I. Atmosukarto, Ben J. C. Quah

**Affiliations:** ^1^ Irradiation Immunity Interaction Lab, Australian National University, Canberra, ACT, Australia; ^2^ Division of Genome Sciences & Cancer, John Curtin School of Medical Research, Australian National University, Canberra, ACT, Australia; ^3^ Australian National University, Canberra, ACT, Australia; ^4^ Department of Medical Oncology, Canberra Hospital & Health Services, Canberra, ACT, Australia; ^5^ Radiation Oncology Department, Canberra Hospital & Health Services, Canberra, ACT, Australia

**Keywords:** cancer, immunology, machine learning, leukocytes, flow cytometry, biomarkers

## Abstract

**Background:**

Machine learning (ML) is a valuable tool with the potential to aid clinical decision making. Adoption of ML to this end requires data that reliably correlates with the clinical outcome of interest; the advantage of ML is that it can model these correlations from complex multiparameter data sets that can be difficult to interpret conventionally. While currently available clinical data can be used in ML for this purpose, there exists the potential to discover new “biomarkers” that will enhance the effectiveness of ML in clinical decision making. Since the interaction of the immune system and cancer is a hallmark of tumor establishment and progression, one potential area for cancer biomarker discovery is through the investigation of cancer-related immune cell signatures. Hence, we hypothesize that blood immune cell signatures can act as a biomarker for cancer progression.

**Methods:**

To probe this, we have developed and tested a multiparameter cell-surface marker screening pipeline, using flow cytometry to obtain high-resolution systemic leukocyte population profiles that correlate with detection and characterization of several cancers in murine syngeneic tumor models.

**Results:**

We discovered a signature of several blood leukocyte subsets, the most notable of which were monocyte subsets, that could be used to train CATboost ML models to predict the presence and type of cancer present in the animals.

**Conclusions:**

Our findings highlight the potential utility of a screening approach to identify robust leukocyte biomarkers for cancer detection and characterization. This pipeline can easily be adapted to screen for cancer specific leukocyte markers from the blood of cancer patient.

## Introduction

Machine learning (ML) has enormous potential to aid in the optimization of clinical decision-making for cancer patient care (1). Although this idea has propelled multiple ML-supplemented healthcare initiatives (2), many of these have failed to reach the standard required to be readily used in clinical practice (3–9). This might be attributable to the previous ML projects being built on a generalized cancer patient population rather than a more defined pool of patients and outcome targets from which more specific data input for ML could be acquired (10). The ideal clinical data for ML model training should be easily and objectively attainable, and reliably correlate with the clinical outcome of interest. This, in turn, highlights the importance of appropriate data selection for ML model training and the that the limits of ML models’ predictive capacity are restricted due to the dataset they were trained on (11). Therefore, to optimize the utility of ML predictive models, it is paramount to identify data, often referred to as “biomarkers”, that robustly correlates with the prescribed outcome.

The last few decades have seen the immune system identified as a central modulator for tumor initiation and progression. While immune cells can recognize and eliminate tumor cells, cancer cells are now understood to escape such surveillance through various immuno-suppressive mechanisms, often through hijacking of the immuno-regulatory framework in the host’s immune system (12, 13). This tug-of-war within the immune system can manifest in cancer-specific immune cell signatures locally in the tumor microenvironment and systemically in the secondary lymphoid tissues and blood (14–17). In this context, we have hypothesized that immune cell profiles, interpreted through ML models, may be predictors of cancer progression and response to treatment. This is supported by our earlier preclinical investigations in murine orthotopic tumor models that identified blood myeloid cell profiles as key predictive biomarker for the presence, type and progression of cancer in ML models (18).

To build further on our approach, we developed a multiparameter cell-surface marker screening pipeline using flow cytometry to obtain high-resolution systemic leukocyte population profiles that correlate with the presence of cancer in our murine syngeneic tumor models. Our rationale was that identification of various changes in the specific, cancer-associated leukocyte populations in blood would allow us to use this data for more robust ML model training for detection and characterization of cancers. We used a screening pool of multiple samples from cancer-bearing and healthy mice that were differentiated by fluorescent vital dye barcoding (19–22). This was then coupled with a leukocyte lineage fluorescent antibody backbone panel and a commercial screen that applies >250 fluorescently tagged antibodies (23) to identify cell surface markers. Using this approach, we were able to effectively screen >20 leukocyte populations in nine distinct samples from cancer-bearing and non-cancer control groups in one pooled sample and identify cancer-specific changes across ~250 markers. From cancer-specific markers that were identified, a cancer-specific antibody panel was designed to characterize the blood leukocyte subsets from 44 mice bearing either no tumor or breast or colorectal cancer by flow cytometry. Using the panel, we identified several leukocyte subset profiles, the counts of which acted as predictive biomarkers for the presence and type of cancer using ML. Our findings highlight the potential utility of a screening approach to identify robust leukocyte biomarkers for cancer detection.

## Methods

### Animals

C57BL/6 (B6) and BALB/c (BC) female mice aged between 6-10 weeks (from the Australian Phenomics Facility, ANU) were used in the study. Animals were housed in a specific pathogen-free environment and used under strict adherence to protocols approved by the institutional Animal Experimentation Ethics Committee (AEEC), ANU, under protocol A2020/39. At experimental end points, animals were euthanized by cervical dislocation according to AEEC approved procedures.

### Cell lines

The mammary carcinoma cell lines 4T1 (24) (ATCC), 4T1.2 (25) (kindly provided by Dr Robin Anderson, Olivia Newton-John Cancer Research Institute), 4T1Br4 (26) (kindly provided by Dr Normand Pouliot, Olivia Newton-John Cancer Research Institute), and AT-3-OVA (27) (kindly provided by Dr. Di Yu), the colorectal carcinoma cell lines CT26 (28) (ATCC) and MC38 (29) (kindly provided by Dr. Di Yu), and the melanoma cell line B16-F10 (30) (kindly provided by Dr Christopher Parish) were used in this study. Cell lines were confirmed clear of specific pathogens by Cerberus Sciences (ISO 9001 Licence No. AU843-QC). Cell lines were cultured and subcultured as described previously in supplemented (sRPMI) RPMI-1640 (11875093, ThermoFisher Scientific) (18).

### Tumor establishment

Tumor cells (1 x 10^5^) were injected subcutaneously in the right hind flank (primary tumor) and then 3 days later in the left hind flank (secondary tumor) of syngeneic mice (cell lines 4T1, 4T1.2, 4T1Br4, and CT26 injected in BC mice, and cell lines AT-3-OVA, MC38 and B16-F10 injected in B6 mice) mixed across housing cages as described previously (18). Tumors were grown and monitored for 17-21 days. At study endpoint, mice were humanely sacrificed, and their tumor and spleens excised and weighed. The tumor burden was defined as the pooled tumor weights of each individual at end point.

### Blood and spleen collection and processing

Blood and spleens from mice were collected at experimental end point. Blood was collected and processed as described previously (18). Spleens were harvested and processed to single cells as described previously with the exception that the red blood cell lysis step was not performed (31).

### Spleen cell barcoding

Spleen cells from 3 (of the 3-5) replicate mice bearing the largest mass of 14-17-day-old tumor from either 4T1, 4T1.2, 4T1Br4, AT-3-OVA, CT26, MC38 or B16-F10 cell lines, or no-tumor controls (host B6 or BC mice) were pooled into 9 separate tubes in a total of 10 milliliters (mL) of phosphate buffered saline (PBS). The 9 spleen cell groups were adjusted to the equivalent of 2 spleen masses based on spleen weights (~equivalent to the mass of 2 normal spleens) by removing appropriate volumes of cell suspension from each tube. Cells volumes were then made to 10 mL with PBS and cells sedimented by centrifugation (300 x g for 10 min), supernatant aspirated and cells resuspended in 2.9 mL sRPMI. Each spleen cell group was then barcoded separately with a unique concentration of carboxyfluorescein diacetate succinimidyl ester (CFSE) and/or cell trace violet (CTV) (**Table S1**) and all groups were then pooled into one sample as previously described (19). Cells were then suspended in 10 mL of sRPMI and passed through a 70 μm filter mesh and counted. A total of 400 x 10^6^ leukocytes was then suspended in 10 mL sRPMI, passed through a 70 μm filter mesh, sedimented by centrifugation (300 x g for 10 min) and supernatant aspirated, ready for immediate backbone antibody labelling.

### Backbone antibody labelling

Barcoded pooled cells were resuspended in 0.6 mL of Labelling Buffer (PBS containing 5 mM EDTA, 1% BSA [*weight/volume*]) with 5 μg/mL TruStain FcX™ (anti-mouse CD16/32) antibody (101320, Biolegend) for 15 min at 4°C. Samples were then incubated with a backbone panel of antibodies (**Table S2**) by adding 0.6 mL of Labelling Buffer with 10% (*v/v*) Brilliant Stain Buffer Plus (BD) containing a 2x stock of each antibody (**Table S2**), for 30 min at 4°C. The pooled barcoded and backbone antibody-labelled cells were then resuspended to 13.4 x 10^6^ cells per mL (i.e. 1x10^6^ cells/75 μL) in Labelling Buffer and passed through a 70 μm filter mesh ready for aliquoting into the wells of the LEGENDScreen plates.

### LEGENDScreen assay

A LEGENDScreen Mouse PE Kit (BioLegend) was used for spleen leukocyte screening for cancer-specific cell-surface markers. Plates from the kit were prepared according to the manufacturer’s instructions with lyophilized antibodies in each well of the assay plates being resuspended in 25 μL of deionized H_2_O (dH_2_O). The pooled barcoded and backbone antibody-labelled cells were added at 75 μL (i.e. 1x10^6^ cell) to each well containing the reconstituted antibodies and incubated in the dark for 30 min at 4^°^C. Cells were then washed in Legend Screen Wash provided in the kit, pelleted and resuspended in 40 μL Labelling Buffer containing 1 μg/ml of the viability dye Hoechst 33285 (Invitrogen) and the equivalent of 500 Flow-Count Fluorospheres (7547053, Beckman Coulter) per 40 μL and stored at 4°C overnight before flow cytometry.

### Immunophenotyping of blood leukocytes by flow cytometry

Blood samples (5 μL) were labeled with antibodies that included the backbone panel and the screen-identified antibodies (**Table S2**) and prepared for flow cytometry analysis using methods described previously (18).

### Flow cytometry

Flow cytometry was performed on a BD LSR-II X-20 (BD Bioscience) flow cytometer with FACSDiva software (version 8.0.1). Application Settings were applied to standardize fluorescence intensity readings between experiments, and fluorescence intensities monitored and calibrated using Sphero™ 8-peak Rainbow Beads (110620, BD Bioscience). Voltages were initially set up using unlabeled RBC-lysed blood leukocytes. BD CompBeads (552843, BD Bioscience) were used as compensation controls as previously described (31). Blood cell samples were acquired until a total of 2000 Flow-Count Fluorosphere beads were collected based on side scatter (log-scale) and forward scatter (linear-scale) plot gating. LEGENDScreen samples were acquired at 10,000 events/second using the sample fine adjust and on a low sample flow rate to collect a total of ~1-3 x 10^5^ live (Hoechst 33285^−^-gated) CD45^+^ cells. Every 36^th^ sample acquisition was followed by a 3 min run on a high sample flow rate with 10% sodium hypochlorite solution in dH2O, then a 2 min run on a high sample flow rate with dH_2_O, and the stability of fluorescence signal of each channel assessed by acquiring 5000 Sphero™ 8-peak Rainbow Beads. Raw Flow Cytometry Standard (FCS) files (i.e., FCS3.1) of the data are available upon request at the ANU DATA COMMONS repository (https://dx.doi.org/10.25911/zrp3-nd51).

### Flow cytometry analysis

Flow cytometry analysis was performed using FlowJo v10 software (BD Bioscience), CytoExploreR version 2.0.0 (32), and cytoverse suite of R packages (33, 34). A combination of manual gating and unsupervised Pairwise Controlled Manifold Approximation Projection (PaCMAP) (35) analysis was used to delineate cell populations and assess for manual gate cell population segregation, and cell groups were then named based on marker expression represented by median fluorescent intensities (MedFI) of each marker plotted using heat map dot plots made using the tidyverse suite of R packages (36) (see Results section).

### Data normalization and processing

#### LEGENDScreen data

Leukocyte marker expression changes in cancer samples were compared to control levels as follows: First, the average median fluorescence intensity of the PE channel (MedFI-PE) from the fluorescence minus one (FMO) controls in the screen for each leukocyte population (identified with the backbone antibody panel) within each barcoded group was calculated. This FMO MedFI-PE was then subtracted from each MedFI-PE from the corresponding barcoded group leukocyte population across all the markers screened to give a barcode corrected MedFI-PE (BC-MedFI-PE). Background (matched no-cancer controls) BC-MedFI-PE of LEGENDScreen markers for each cell population was subtracted from the corresponding marker BC-MedFI-PE of the same cell population in each tumor type. This BC-MedFI-PE difference was then divided by the maximum BC-MedFI-PE change of each marker for each population. Any value less than -1 was assigned -1. This gave a cancer-specific marker change scale of -1 to 1 (with 0 being no-cancer controls level). These values were visualized on a heat map dot plot using the R package ComplexHeatmap (37). The raw and normalized MedFI data and the data for cell counts and proportions for heatmap annotations can be found in **Supplementary Data (Data Sheets 2, 3)**.

#### Blood leukocyte data

To reduce the influence of inter-experimental technical variability on the independent blood analysis experiments, their data was normalized at several levels. Firstly, cell numbers in each flow cytometry acquisition set were normalized to counting beads spiked into the sample, with each sample normalized to 5000 Flow-Count Fluorospheres (all of the spiked load), to give the number of cells in ~5 μL of blood (“counting bead normalized” values). Secondly, these normalized counts were normalized to the mean counts of the respective blood leukocytes from non-tumor bearing control animals within each experiment, the “nil normalized values”. To get “normalized cell counts” per 5 μL of blood (as an estimate of the overall cells across the groups), the “nil normalized values” were multiplied to the overall mean of the “bead normalized cell count” from all non-tumor-bearing animals for each cell population across all experiments. This data can be found in **Supplementary Data (Table 1)**.

### Supervised machine learning

Supervised machine learning was performed using Orange 3 software. Random Forest (38) and CATboost (39) modelling used 100 trees for prediction and 500 trees for ranking the feature importance, with the maximum tree depth of 4 (for Random Forest) and 6 (for CATBoost). For Random Forest, the maximum number of features considered at each node was 5 and subsets smaller than 5 were not split. For CATBoost learning, the learning rate was 0.3, the regularization was lambda 3 and subsampling was 1. For classification of groups using monocytes, CATBoost was used and trained on 66% of randomly sampled data and tested on the remaining 34%. This was repeated 100 times and the results of predicted and actual classes were displayed as a confusion matrix. Feature ranking was done using both Random Forest and CATBoost (built into the models in Orange 3 software). For the learning curve as a function of decreased features (populations), CATBoost was used and trained on 66% of randomly sampled data and tested on the remaining data. This was repeated 100 times and the results were assessed using area under curve of the receiver operating characteristics (AUC; to assess separability of the classes), classification accuracy (CA; proportion of correct classification), precision (ratio of correct positive prediction to all positive prediction), recall (ratio of correct positive prediction to actual positive), and F1 score (weighted average of precision and recall). Final CATBoost prediction on the optimized feature subset was trained on 66% of randomly sampled data and performed on the remaining data.

### Statistical analysis and data presentation

To compare the means between non-tumor controls (Nil), CT26- and 4T1-burdened cohorts, data were transformed using the formula Y=Log(Y+1) to help normalize distributions and equalize variance, and then assessed by 2-way ANOVA using GraphPad Prism software. Analysis was corrected for multiple comparisons using the two-stage step-up method of Benjamini, Krieger and Yekuyieli (40). False discovery rate of 0.05 and p-values were reported to test the null hypothesis that the means are equal or distributions were from the same population. The overlap R package was used to calculate distribution overlap (41). PaCMAP used the pacmap python package through CytoExploreR. Multidimensional scaling was performed using the cmdscale function from the stats (v3.6.2) R package to visually inspect the improvement in group separability when running ML models using the highest ranked populations. Heatmap dot plots were generated through ggplot2, ComplexHeatmap and HeatmapR (42) R packages. Log ratio (M) log average (A) (MA) plots were constructed using the ggpubr, ggplot2 and ggrepel R packages. Pythagorean trees and confusion matrices were made in Orange 3 software. Circular bar plots were made using ggplot2 in R. The R version used was 3.6.2. Prism was also used for plotting data.

## Results

### Screening pipeline overview

**Figure 1:**
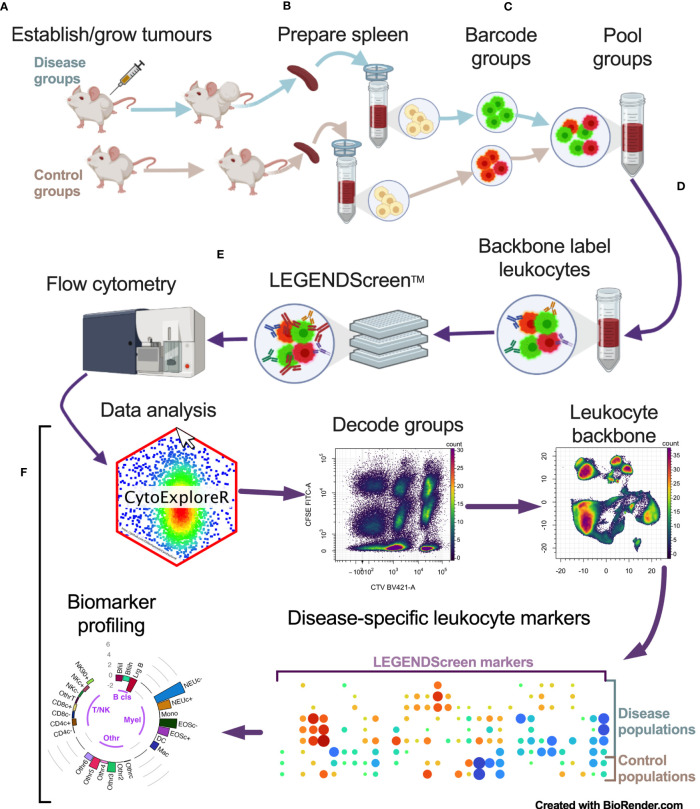
Multiparameter cell-surface marker screening pipeline overview. Tumor-bearing and no-tumor control groups had spleens (and/or blood) harvested and prepared as single cells **(A, B)**. Single cells were barcoded with vital dyes to allow for fluorescent discrimination and were then pooled **(C)**. Pooled cells were labeled with a backbone of antibodies to delineate the main leukocyte subsets **(D)**. Cells were then aliquoted equally across the wells of the LEGENDScreen plates for PE-conjugated antibody labeling and analyzed by flow cytometry **(E)**. Data was then analyzed by decoding barcoded groups, delineating leukocytes based on backbone antibody labeling, and assessing for disease-specific changes in LEGENDScreen markers to identify new leukocyte phenotyping markers for profiling biomarkers for cancer presence **(F)**.

To identify cancer-specific leukocyte surface marker changes, a screening pipeline was established (**Figure 1**). This involved: a), establishing tumors in mice; b), preparing spleen cells from tumor-bearing mice and their non-tumor-bearing counterparts: c), fluorescent barcoding of the spleen cells from each group with distinct concentrations of CellTrace Violet (CTV) and/or carboxyfluorescein succinimidyl ester (CFSE) vital dyes to give distinct fluorescent groups; d), pooling the barcoded cells and labeling them with a backbone of lineage marker-specific antibodies to identify the main leukocyte populations; e), aliquoting the pooled, barcoded and backboned samples equally into wells of LEGENDScreen assay plates to label them with the >250 PE-conjugated antibodies specific for cell-surface markers for analysis by flow cytometry; and f), assessing the samples for cancer-specific changes via an analysis pipeline.

### Tumor models and initial barcode/backbone analysis

**Figure 2:**
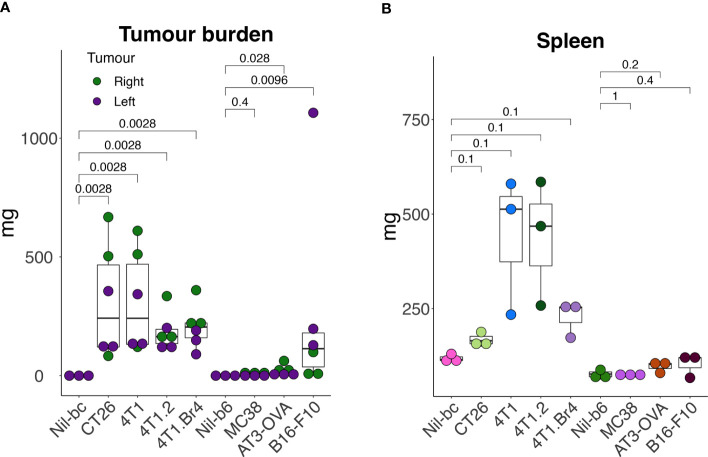
Tumor and spleen masses in animals used in the screening pipeline. Tumors were established subcutaneously in the right- (day-0) and in the left- (day-3) hind flank of syngeneic mice [cell lines 4T1, 4T1.2, 4T1Br4, and CT26 injected in BALB/c mice (BC), and cell lines AT3-OVA, MC38 and B16-F10 injected in C57BL/6 mice (B6)] and grown for 17 days. At endpoint, tumors **(A)** and spleens **(B)** were excised and weighed. All data points are presented with overlayed boxplots from n = 3-5 mice. P-values are shown comparing means of data from tumor-bearing mice with their no-tumor control (Nil) counterparts. To compare the means between non-tumor controls (Nil), CT26- and 4T1-burdened cohorts, data were transformed using the formula Y=Log(Y+1) to help normalize distributions and equalize variance (to meet the assumptions of the statistical tests), and then assessed by 2-way ANOVA.

To assess how the leukocyte subpopulation profiles might change in the presence of cancer, seven murine tumor cell lines representing breast (including, 4T1 (24), 4T1.2 (25), 4T1Br4 (26), AT-3-OVA (27)), colorectal (including CT26 (28) and MC38 (29)) and skin (including B16-F10 (30)) cancers were used to establish tumors in syngeneic strains of mice (B6 and BC), yielding a total of nine groups (seven cancer and two no-cancer control samples). A primary and secondary tumor was established on each hind flank (left and right; to mimic disseminated disease) of each animal for each cell line and grown for 17 days. The endpoint masses showed variable degree of growth, with the tumors on the BC background typically weighing more than those on B6 background (**Figure 2A**). Although most animals had viable tumors, MC38 tumors had negligible mass at the end point (**Figure 2A**). The weight of the spleens extracted from these mice for the LEGENDScreen pipeline also varied considerably between groups, lending support to the theory that certain tumor types may promote spleen enlargement (38) (**Figure 2B**).

**Figure 3:**
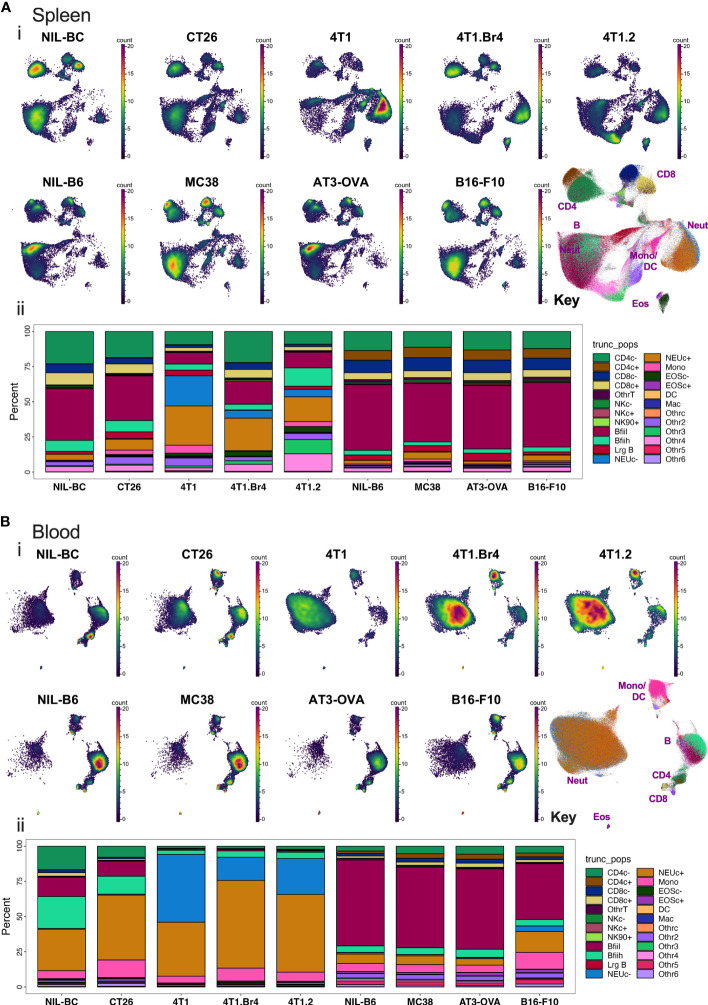
Spleen and blood leukocyte frequencies determined using backbone panel markers. Spleen **(A)** and blood **(B)** cell samples were analyzed as described in **Figure S2** and assessed for changes across the different tumor-bearing and no-tumor control groups. In upper panels (i): PaCMAP plots for each group are shown for 50,000 live singlet CD45^+^ leukocytes per plot with a PaCMAP key to show subpopulation position with color overlays delineating each population present from spleen **(A)** or blood **(B)**. In the lower panels (ii) of **(A, B)**, the percentage of total live singlet CD45^+^ leukocytes comprising each live singlet leukocyte subpopulation was also calculated and shown below the respective PaCMAP plots. For spleen, the delineation of each group was achieved by decoding the barcoded groups based on manual gating (**Figure S1**).

After processing the leukocytes through the analysis pipeline, we investigated how the standard lineage backbone antibody labelling characterized the immune cells in the pooled spleens and blood of the animals. The backbone panel comprised primarily antibodies to delineate key leukocyte subsets. Manual gates were created to identify ~24 main cell subsets (**Figure S1**). The general population segregation was confirmed by overlaying the manually gated populations on graphs of unsupervised Pairwise Controlled Manifold Approximation Projection (PaCMAP) dimensional reduction of all the backbone markers in 2-dimensional space (**Figures S2A, B**). The marker expression of the segregated populations was summarized by dot plot heat maps annotated with pooled cell proportions (**Figure S2C**) and used to confirm population name designations (**Table S3**). Manual gates were also used to delineate the barcoded groups (**Figure S1**), and the proportion of CD45^+^ live leukocytes comprising each of the identified leukocyte subpopulations was calculated across the groups (**Figure 3**). Major changes were seen in the leukocyte composition of all animals with tumors on BC backgrounds in both spleen and blood, whereas more a subtle change in the leukocyte composition was observed in animals with tumors on a B6 background. The major tumor-specific changes appeared to be the proportional increases in myeloid cells, most notably monocytes and neutrophils, particularly in BC mice burdened with 4T1 tumor variants.

### LEGENDScreen global changes

To resolve the leukocyte subpopulation differences at higher resolution, we assessed each of the backbone antibody panel-identified splenic leukocyte populations across all tumor groups for changes in the median fluorescent intensities (MedFI) of the >250 PE-conjugated LEGENDScreen antibodies (herein, termed MedFI-PE) relative to background (no tumor) cell levels (**Figure S3**). This was summarized in a dot plot heat map where an increase in the dot size reflected an increase in the absolute MedFI-PE change in tumor-bearing animals relative to the background control (either increase or decrease). In addition, the plots showed increases and decreases in the relative marker MedFI-PE from the background by the dot color tending toward darker red or darker blue, respectively (**Figure S3**). This global analysis demonstrated that while most of the cell surface markers showed little change in the presence of cancer, certain leukocyte population markers underwent clear changes across several tumor types (**Figure S3**).

### LEGENDScreen CT26 and 4T1 changes

**Figure 4:**
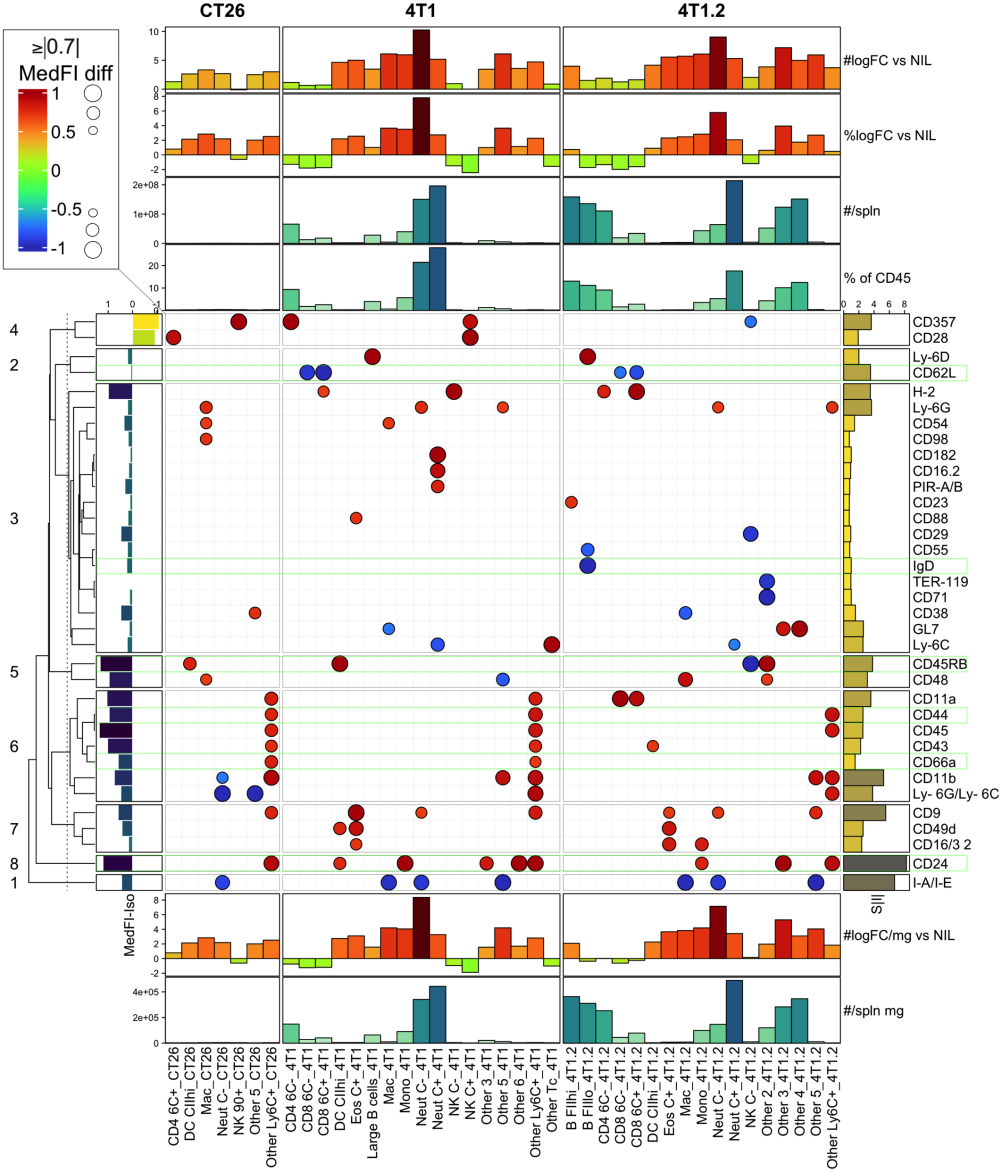
LEGENDScreen surface marker changes in leukocytes from CT26, 4T1 or 4T1.2-bearing animals. Leukocytes from tumor-bearing and no-tumor control animals (as described in **Figure S2**) were analyzed by the LEGENDScreen pipeline as described in (**Figure S3**). Changes in leukocyte surface marker expression from CT26, 4T1 or 4T1.2-burdened animals relative to background no-tumor controls were plotted as a heatmap dot plot with dot size reflecting absolute change in MedFI-PE (scaled -1 to 1; being 0=unchanged, -1 to 0=decrease, 0 to 1=increase, relative to no-tumor counterpart control) and color reflecting an increase or decrease in MedFI. Markers in the top absolute 30% changed from no tumor background controls are shown. Hierarchical clustering using Euclidean distance was used to group the markers and the relationships summarized using a dendrogram. The 8 most related marker clusters were partitioned and labeled. The expression of each marker was also plotted after subtracting the MedFI-PE of a matched isotype control antibody from its average MedFI-PE (left bar plot annotation). The sum of all absolute MedFI-PE changes (S|I|) was also calculated across the groups for each marker (right bar plot annotation). Finally, enumeration of each CD45^+^ leukocyte subpopulation based on either % of total CD45^+^ leukocytes or total cell number (#) was calculated per spleen (top annotations) or per milligram (mg) of spleen (bottom annotations) and included log2 fold changes (logFC) from no-tumor control (Nil) levels. Results are from pooled samples of n=3 per group.

To narrow the scope of the LEGENDScreen data and inform the next step in the experimental validation, our analysis focused on two cancer types with consistent growth and diverse immune perturbation. These were the CT26 colorectal cancer model, which generated relatively mild immune perturbation, and the 4T1 breast cancer model, which generated marked immune perturbation (18). In addition, markers associated with metastatic potential were assessed using the 4T1 metastatic variant, the 4T1.2 line (25) (**Figure 4**). To focus on the strongest cancer-associated marker changes, a threshold was set to display markers that were within the top 30% of the increased or decreased MedFI-PE change relative to background no-tumor controls (**Figure 4**). This was an arbitrary range set to filter for the most extreme changes in surface marker labeling. For quality control, the expression of each marker after subtracting the MedFI-PE of isotype control antibodies (**Figure 4**, left bar plot) was also determined, which revealed that all markers had a positive signal above matching isotope control binding except CD357 and CD28 markers, which had negative fluorescence relative to their isotype antibody controls, suggesting a technical anomaly. CD357 and CD28 markers were thus excluded from further analysis. Among the remaining surface markers, CT26 tumors clearly induced fewer marker changes than 4T1 tumors (**Figure 4**). While the tumors from 4T1 and their metastatic variant 4T1.2 resulted in a similar number of marker changes, some of these were clearly variant-specific, potentially relating to the metastatic tendency. Since only up to 6 additional antibodies could be added to the backbone antibody panel for further studies given the flow cytometer instrumentation restrictions, the sum of all absolute MedFI-PE changes was calculated across the groups for each marker to identify those with the greatest cumulative changes (**Figure 4**, right bar blot). To ascertain the cell population numbers associated with marker changes, enumeration of each cell population based on % of total CD45^+^ leukocytes and total leukocyte population per spleen (**Figure 4** top annotations) and cell leukocyte population amounts per milligram of spleen (**Figure 4** bottom annotations) were also calculated. These demonstrated that CD24, CD45RB, and CD44 had relatively strong changes across all tumor types, rendering them the preferred candidates for panel inclusion. Also notable was CD62L downregulation on CD8^+^ T cells in 4T1 and 4T1.2, but not CT26, models; CD66a upregulation on myeloid subpopulations in 4T1 and CT26, but not 4T1.2, models; and surface IgD downregulation on B cells in the metastatic 4T1.2 model but not its parental line, 4T1. This combination of markers therefore might reflect the changes that are broadly associated with cancer presence, specific to cancer type and its metastatic potential. While there were several other marker changes in the models, many of these were already in the backbone panel, and others, while potentially of value, were not included in the expanded labelling panel due to restrictions on the total number of markers based on flow cytometry infrastructure. The expression of these markers on the leukocyte populations in which they underwent the most apparent change was also displayed in raw histogram format (**Figure S4**). This demonstrated that the degree of the changes ranged from subtle to obvious between the CT26, 4T1 and no-tumor control groups.

### LEGENDScreen informed leukocyte-profiling in CT26 and 4T1 bearing animals

**Figure 5:**
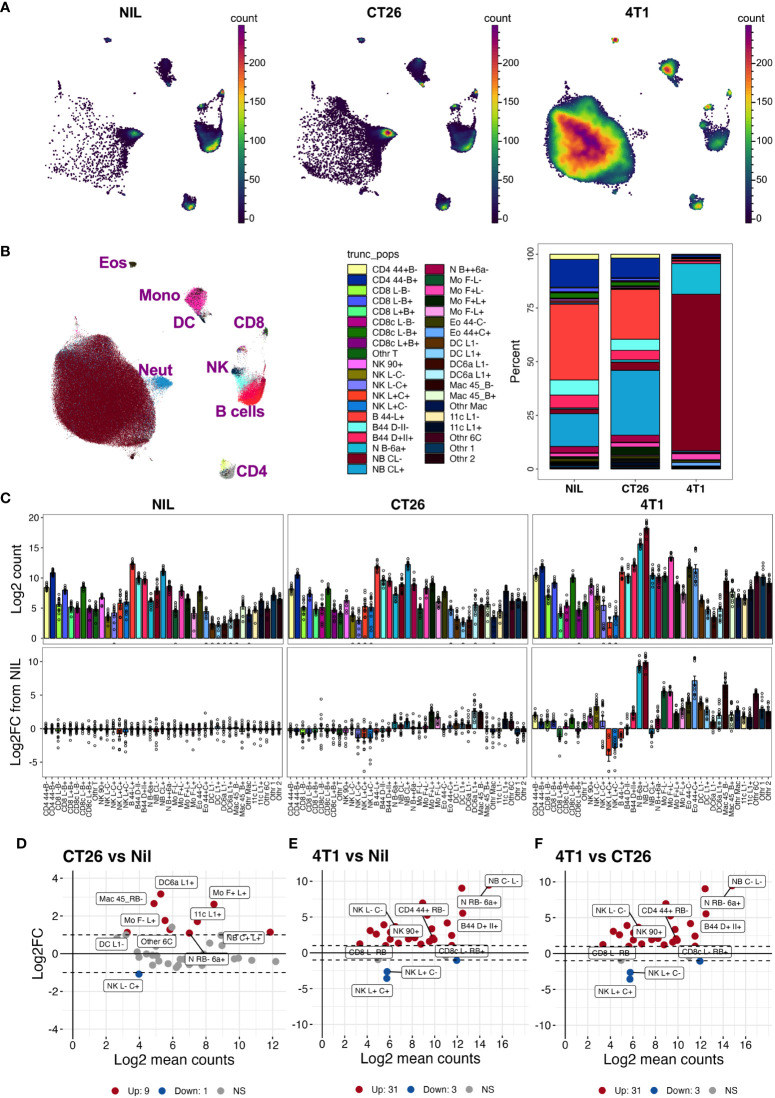
Blood leukocyte subset changes due to cancer presence. Blood leukocyte populations from CT26- and 4T1-bearing mice and no-tumor controls (Nil) were identified as described in **Figure S5**. Live singlet CD45^+^ leukocytes from each group were analyzed using PaCMAP dimensional reduction of a total of 50,000 cells per group collated from 5 separate mice per group **(A)**. Leukocyte populations were identified by manual gating (**Figure S5**) and overlaid in a reference PaCMAP plot of collated groups [**(B)**, first panel]. Leukocyte subset compositions were calculated by dividing subpopulation counts by total number of live CD45^+^ leukocytes for each group [**(B)**, second panel)]. Total numbers of leukocytes per 5 μL of blood were calculated as described in the Methods and presented as log2 count [**(C)**, top panels]. Fold changes of each population in cancer-bearing animals over the mean levels of no-tumor controls were also calculated [**(C)**, bottom panel]. Log ratio (M), log average **(A)** (MA) plots showing log2 average counts per 5 µL of blood against log2 fold changes in counts were plotted for each pairwise group comparison and statistically significant leukocyte count changes in the groups colored red for upregulated (Up) counts or blue for downregulated (Down) counts relative to the no-tumor reference group [Nil **(D, E)** or CT26 **(F)**]. The nine highest ranked leukocyte count changes, based on smallest p value, are labelled. Dotted lines indicate 2-fold changes (2 FC). Samples were from a total of 15 animals per group, except for the 4T1 group which had 14 (due to the loss of one replicate), spread across 3 independent experiments. Statistical significance was assessed using two-way ANOVA on Log10(Y+1)-transformed data corrected for multiple comparisons using the two-stage step-up method of Benjamini, Krieger and Yekuyieli (37) and a false discovery rate of 0.05.

With the inclusion of CD24-, CD45RB-, CD44-, CD66a-, CD45RB-, and IgD-specific antibodies to the existing leukocyte-profiling backbone panel, we analyzed the blood leukocytes from animals bearing CT26, 4T1 or no tumors across three independent experiments. While the tumor growth was variable, all mice in the CT26 and 4T1 groups had palpable tumors by the endpoint (**Figure S5A**). Manual gating was used to identify leukocyte populations across all experimental groups (**Figures S5B, C**); gated populations overlaid onto a 2-dimensional PaCMAP embedding (**Figures S6A, B**); and phenotype assessed on a dot plot heat map displaying MedFI of marker expression and population proportions (**Figure S6C**). Up to 39 populations were identified with the inclusion of the additional antibodies (**Figures S6B, C, Table S4**). Separating the animal groups into no-tumor control (Nil), CT26 and 4T1 models (**Figures 5A, B**) showed obvious proportional (**Figure 5B**) and total count (**Figure 5C**) changes in the delineated blood leukocyte populations across the groups. There were obvious fold changes in the counts of numerous leukocyte subsets in both tumor types relative to normal levels, which was more pronounced in animals with 4T1 tumors (**Figure 5C**). The counts of nine populations of myeloid cells were significantly increased by >2-fold above control in CT26 samples (**Figure 5D**); the increases were more dramatic in 4T1-bearing mice with most myeloid cells showing a significant >2-fold increase above no-tumor control and CT26-bearing animals (**Figures 5E, F**). In addition, the 4T1-bearing mice had significant >2-fold increases in most counts of lymphoid populations compared to the other groups (**Figures 5C, E, F**). In both tumor models, NK cell subpopulation counts were significantly decreased at >2-fold levels compared to the no-tumor control animals, and 4T1 also showed a decrease in CD62L^+^ CD45RB^+^ CD8^+^ T cells compared to the other groups (**Figures 5D–F**). This illustrates the wide scope of the blood leukocyte count changes and their complexity in relation to the cancer growth, which also highlights the utility of adding markers to identify the leukocyte population changes based on a screening approach.

### Finding cancer-defining leukocyte biomarkers by assessing distribution overlap

**Figure 6:**
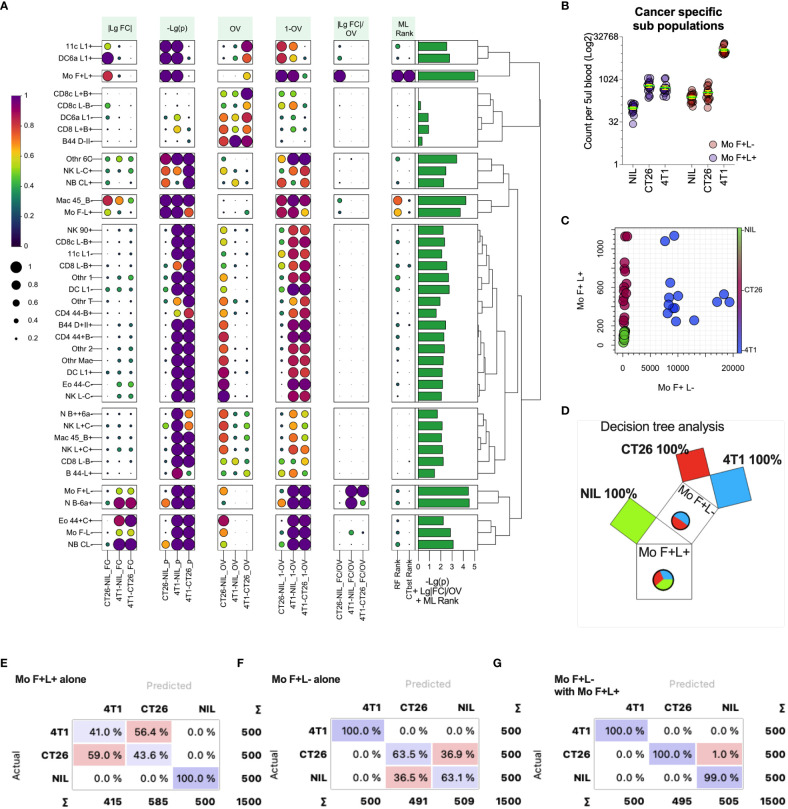
Metrics for identifying blood leukocyte subsets to delineate groups. Blood leukocyte population counts from CT26- and 4T1-bearing mice and healthy no tumor controls (Nil), were identified as described in **Figure 5**. In plot **(A)** leukocyte counts were compared pairwise between the groups (as indicated along the x-axis) to generate several statistics including: fold change of count means, represented as absolute value of the log2 of the fold change (|LgFC|); adjusted p values to test the probability the distributions are from the same population, represented as the -log2 of the p value (-Lg(p), i.e. the larger the value the smaller the p-value); the proportion of overlap of the distributions (OV) which was also represented as the 1-OV (i.e. the larger the value the less overlap); and the fold change divided by proportion of overlap represented as log2 of the absolute fold change divided by OV (|LgFC|/OV). In addition, the rank of importance (ML Rank) of each leukocyte population from Random Forest (RF Rank) and CATBoost (CTbst Rank) ML models in classification of the groups was displayed. A cumulative group discriminating score for each leukocyte subset was generated by adding -Lg(p), Lg|FC|/OV and ML ranks together, which is displayed as a right bar plot annotation. The subsets were organized in 9 groups based on Euclidean distance hierarchical clustering across all metrics summarized as a dendrogram. Each metric was scaled from 0 to1 (from low to high) for each pairwise group comparison or ML rank except for -Lg(p), which was scaled across all comparisons. Plot **(B)** shows the blood cell counts of the two monocyte subsets with least overlap between the groups. Plot **(C)** shows the relationship of the counts of the two monocyte subsets for each individual highlighting group segregation. Plot **(D)** shows a Random Forest-based decision Pythagorean tree (43), which shows the relative amount of classified individuals as box volume, purity based on color (both of which are summarized by pie charts, being Nil = green, red = CT26 and blue = 4T1) and the subpopulation used to split the data at each node. Plots **(E-G)** show confusion matrices for group predictions using CATboost ML models. CATBoost and Random Forests models used 100 trees and were trained on 66% of randomly sampled data and tested on the remaining data and this repeated 100 times using non-replicable training.

While fold changes and p values can help test the group similarities in leukocyte counts, we sought to generate a statistic that reflects the populations that are most capable of distinguishing between the tumor-bearing and healthy groups. This would be particularly useful in identifying the blood cell biomarkers that correlate with the presence and type of cancer in a clinical setting. For this, we assessed pairwise the measures of differences in the blood leukocyte population between CT26- and 4T1-bearing and no-tumor groups, including the metrics based on standard fold changes (**Figure 6A**, |LgFC| column) and p-values (**Figure 6A**, -Lg (p) column), but also an overlap statistic (41) to measure the overlap of the blood count distributions of a given leukocyte population between groups (**Figure 6A**, OV & 1-OV columns). The rationale here is that the distribution of a particular leukocyte count that showed the least overlap between groups would be better at distinguishing the groups. Leukocyte counts with the largest fold change and smallest overlap between groups would be an ideal biomarker for distinguishing cancers and could be presented as a single statistic, with the ratio of fold change divided by overlap (**Figure 6A**, |LgFC|/OV column). From this analysis, the top distinguishing population counts appeared to be from the monocyte subsets that had large |LgFC|/OV values (**Figure 6A**). Among these monocyte populations, the counts of monocytes that expressed higher levels of CD62L (Mo F+L+; monocytes expressing SiglecF and CD62L) had large fold changes and small p-values (larger -log2(p)) between the groups, and notably had minimal count overlap between the tumor-bearing groups and no-tumor controls, thus representing a suitable marker to distinguish cancer presence. In addition, monocytes that downregulate CD62L (Mo F+L- monocytes expressing SiglecF and lower levels of CD62L) also displayed similar statistical trends, but these were for distinguishing 4T1-bearing mice from both CT26-bearing and no-tumor control mice (**Figure 6A**). These two monocyte populations, therefore, harbor the potential to distinguish all groups. Indeed, plotting the counts of these populations (**Figures 6B, C**) showed a clear pathway to distinguish no-tumor animals from cancer-bearing animals and also differentiate between the two cancer types based on a decision tree analysis (**Figure 6D**). To highlight this further, these two monocyte populations were used alone or together to train and test a decision tree ML model to assess the capacity to predict the presence and type of cancer from blood leukocyte profiling (**Figures 6E–G**). The counts of monocytes expressing higher levels of CD62L (Mo F+L+) could classify the presence or absence of tumors but could not distinguish the tumor type (**Figure 6E**). The counts of monocytes expressing lower levels of CD62L (Mo F+L-) could distinguish 4T1 tumor from the other groups (**Figure 6F**). Using both monocyte populations, all groups could be distinguished (**Figure 6G**), highlighting the utility of overlap statistics in rapid identification of the group-delineating populations.

### Finding cancer defining leukocyte biomarkers using machine learning models

**Figure 7:**
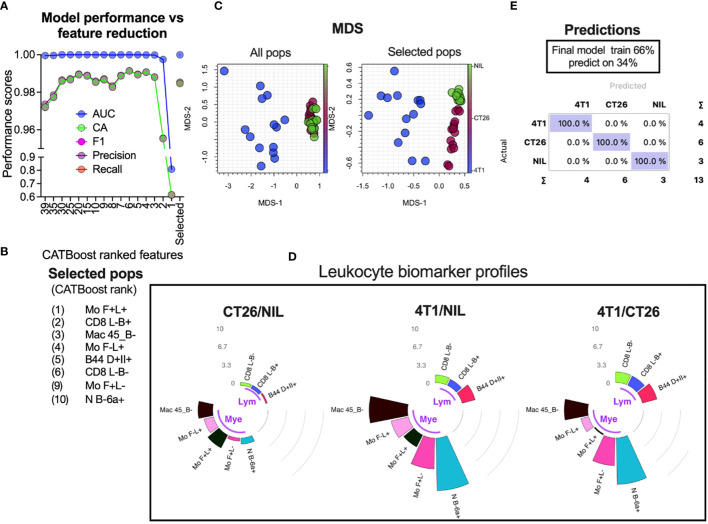
Selection of leukocyte subsets whose counts act as a biomarker for specific cancer detection. Blood leukocyte population counts from CT26- and 4T1-bearing mice and no-tumor controls (Nil), were obtained as described in **Figure 5**. CATBoost ML models were trained on decreased leukocyte subsets based on their CATBoost importance rank in group classification (as shown in **Figure 6A** CTbst rank) or on a selected group of 8 populations (the top 6 CATBoost ranked, and the top 2 cumulative ranked populations based on [as shown in **Figure 6A** right bar plot annotation)] **(A)**. These 8 populations (Selected pops) are listed in **(B)**. The selected populations were compared to all leukocyte populations via MDS to assess how the groups’ distances changed **(C)**. The final CATBoost model was trained on the counts of the selected 8 populations using 66% of randomly sampled data and tested on the remaining data **(D)**. A summary of the selected population blood count log2-fold changes (gray counts and arcs) between the groups is shown in circular bar plots highlighting the lymphocyte (Lym) and myeloid cell (Mye) population changes and illustrates their utility as a collective cancer biomarker **(E)**.

Overlap statistics measure differences in distributions pairwise, and therefore would be best suited to identify key distinguishing features between a few groups. In cases where larger number of groups are being defined or they have larger distribution overlap, more complex models might be required. To establish a generic approach for detecting leukocyte population counts that are most useful for group classification, we used a decision tree-based feature ranking built in the Random Forest and CATBoost tree-based ML models (**Figure 6A** ML rank column). While the monocyte population remained one of the highest ranked features according to the model ranking, several other leukocyte populations of significance were also identified. A cumulative ranking profile based on Random Forest rank, CATBoost rank, p values and the fold-change over overlap ratio was generated to illustrate the overall feature importance across the measures (**Figure 6A** right bar plot annotation). To narrow down the most important features, we created a learning curve to score the classification performance as a function of diminishing leukocyte groups added to the model, and removed the lowest CATBoost ranked populations first (**Figure 7A**). As can be seen, the model performed above 95% of all performance measures with 3 or more top-ranked features. However, there appeared to be variability in performance depending on the number of features, wherein the performance dropped with a large number and enhanced from 8 to 6 top-ranked populations, suggesting that the top 6 features (**Figure 7B**) might be the most informative. Of note, these 6 populations did not include monocyte (Mo F+L-) or neutrophil (N B-6a+; neutrophils expressing low levels CD45RB and higher levels of CD66a) populations that scored the highest on our cumulative ranking profile (**Figure 6A** right bar plot annotation). Given that these two populations are potentially key leukocyte biomarkers for group segregation, we added them to the top 6 CATBoost ranked populations, making a total of 8 populations consisting of various monocyte, neutrophil, CD8^+^ T cell and B cell subsets (**Figure 7B**). To better understand how these 8 populations may perform in cancer classification in one another’s presence, they were compared against all populations for their segregating capacity by multidimensional scaling (MDS) analysis aimed at summarizing the metric distances across all of the 8 leukocyte subsets in a 2-dimensional space (**Figure 7C**). This demonstrated that the selected 8 populations improved group separation relative to all populations, particularly of the CT26 from the no-tumor control (Nil) groups. Indeed, the final CATBoost model trained with the blood counts of these 8 leukocyte populations yielded definitive prediction of all 3 groups (**Figure 7D**). A summary of how the counts of each population change across the groups and act as a collective biomarker profile is displayed as their log2 fold change relative to one another (**Figure 7E**). This clearly illustrates the differential changes in the myeloid (annotated Mye) and lymphoid (annotated Lym) populations in different types of cancer. Thus, our screening pipeline approach has allowed for identification and selection of cancer-specific leukocyte biomarker signatures that are predictive of the cancer presence and its type from a single blood sample.

## Discussion

Accurate classification and staging of cancer is fundamental to its prognostication and management. Conventionally, these have been facilitated by histological investigations and the TNM (tumor-node-metastasis) staging system for over 70 years (44). It is clear, however, that cancers are highly heterogeneous and patients with the same cancer and stage could have widely varying disease trajectories (45). Novel biomarkers that can predict the disease and treatment outcome in each individual would enable highly personalized disease management. ML approaches have the potential to realize such goals by utilizing complex biomarker phenotypes (1). Our study has established a fundamental platform for discovering cancer biomarker patterns through ML-based decision-making processes in a pre-clinical setting. Our tailored pipeline has focused on linking the presence of cancer and its type with the immune cell phenotypes in subcutaneous mouse cancer models, and has characterized several leukocyte markers that are altered in the presence of specific tumors. These correlations may be used to subset “cancer-specific” leukocyte population profiles. In our study, monocyte counts with differential CD62L expression were strongly associated with specific cancers, and populations of neutrophil, T cell, NK cell and B cell subsets were dramatically altered in tumor-bearing animals; these were then used to train the ML model by the pipeline-identified leukocyte subset counts from as little as 5µl of blood. The trained ML models were able to predict the presence and type of cancer with high certainty within the data constraints, suggesting that this approach has utility.

Our pipeline offers several distinct advantages for biomarker discovery. Firstly, it can be easily adapted for use in clinical practice as it focuses on the systemic leukocyte changes from a single draw of blood. Collection of a serum biomarker is less invasive and more cost-effective than a tissue biopsy, suitable for longitudinal monitoring of the disease trajectory, and harbors a wealth of information for biomarker discovery (46–48). Secondly, our screen has utilized fluorescent barcoding to differentiate between the diseased and non-diseased control group samples that are pooled together (19–22), which reduces the inter-sample labeling error rate and acquisition variability. It is also more resource- and labor-efficient as it allows large screens to be performed in a shorter timeframe and enhances analysis workflow by enabling direct comparison between the controls and test samples (19–22, 49). Thirdly, due to the multiplex nature of flow cytometry, our pipeline allows for extensive initial leukocyte phenotyping using a backbone of lineage-defining fluorescently-conjugated antibodies. This, in turn, facilitates the screen to focus on further cell subsets at higher resolution. Importantly, the screening pipeline can be easily adapted to analyze human blood cell subsets by altering the backbone antibody panel and the LEGENDScreen kit, similar to the techniques that are already in use in mass cytometry (50). Our screen could be improved further by multiplexing the screening antibodies within one sample rather than conjugating each screening antibody to one fluorochrome (in this case PE) in separate wells/samples. This would allow higher screening throughput and facilitate multiplex phenotyping, which is the major benefit of single cell flow cytometric analysis. To achieve this, several fluorescently distinct screening antibodies could be grouped into a single well and/or the analysis could be supplemented with modern ML techniques to impute marker co-expression across all the screen markers, as recently reported through a pipeline termed InfinityFlow (51).

The premise of our screen is that the blood leukocyte phenotypes undergo specific alterations as tumors develop and grow. Our study has used subcutaneously injected cell lines to form tumors which replicate established tumors. They do not replicate natural vascularization, metastasis, architecture, immune infiltrate, or growth of spontaneous human tumors (52). However, our study highlights that the pipeline can identify blood leukocyte changes that associate with tumor presence and type, suggesting the pipeline has potential for adoption in human patients. This is in keeping with the established evidence that cancer progression in human patients can result in significant systemic leukocyte changes (53). Such changes are thought to be a consequence of cancer-induced inflammatory responses that play critical roles in tumor initiation, promotion, progression and metastasis while evading immune-surveillance (54). The resultant perturbation in many leukocyte subsets/progenitors such as the changes in the hematopoietic stem cells, dendritic cell phenotype, T cell subset function and NK cell functions, as well as increase in immature monocyte and neutrophil counts and regulatory T and B cells, are thought to be crucial features of cancer (53). Our study supports the potential utility of cancer-induced, ubiquitous blood leukocyte changes as informative biomarkers for disease surveillance. Various populations of blood leukocytes across multiple lineages were altered in our cancer models, with the predominant increase being in the myeloid cell subsets.

In line with our observations, there have been several studies examining the standard full blood counts (FBC) for cancer-specific alterations (46, 53). These studies reported correlations between the neutrophil-to-lymphocyte and monocyte-to-lymphocyte ratios with the cancer treatment outcomes (46, 53). While the standard clinical FBC are currently available for disease outcome prediction in ML models, they might not account for the phenotypic alterations involved in many of the potential leukocyte changes. Techniques using tailored multi-parameter approaches to leukocyte phenotyping such as our flow cytometry-based pipeline enable examination of the leukocyte phenotypes at much higher resolution. Indeed, many of the main leukocyte markers detected in our pipeline to be altered in the presence of cancer have clear functional roles. For example, downregulation of CD62L (55) and upregulation of CD44 (56) are well-known markers for leukocyte activation and have important roles in cell trafficking. IgD, another marker from our pipeline, is expressed on naïve B cells and functions as an antigen-specific B cell receptor for cell activation while also modulating B cell anergy (57). The other pipeline-identified markers CD45RB (58), CD66a (59, 60) and CD24 (61) are also known to modulate leukocyte signaling, and their expression levels may be associated with the functional state of leukocytes. Interestingly, the two monocyte populations that were most selective in separation of our cancer and control groups were delineated by CD62L. The cancer models used in this study were typified by significant increases in the counts of monocytes expressing higher levels of CD62L. Monocytes have diverse roles in tumor development (62), both directly and indirectly as the source of tumor-associated macrophages or dendritic cells, and phenotypically align with monocyte-myeloid-derived suppressor cells that are thought to play key roles in tumor progression (63) Intriguingly, a recent report has identified CD62L^+^ monocytes being recruited to inflammatory sites through high endothelial venules (64) that are common in cancer vascular networks (65), and several reports have identified changes in the monocyte CD62L expression in inflammatory disease states (66–68). Therefore, one could speculate that these cells may influence tumor progression via modulation of inflammatory responses (69, 70), which renders them a potential target for future therapies.

One downside of phenotyping cells using functional markers is that the changes in their marker expression are often detected as cell population shifts rather than splits into distinct sub-populations. Delineating functional cell subsets based on such marker changes may be more subjective and potentially prone to inter-experimental variability, whereas an ideal biomarker should be easily and objectively obtainable. In order to reduce the subjectivity, we incorporated a pre-defined control (no-tumor) sample in each analysis as a reference. However, this approach becomes less practical in the clinical setting due to the inherent heterogeneity of the human population compared to the homogeneous animal clones that were used in our pre-clinical study (71). To improve the clinical translatability, increasing efforts are being made for careful standardization of protocols across institutes, including machine calibration with standardized fluorescent particles, frozen cell normalisation standards, and standardization of sample handling and processing to reduce the technical variability. Encouragingly, evidence to date suggests that this approach is feasible (72, 73). To improve translatability, the number of clinical samples required for ML training will no doubt be much higher than that collected in animal clonal models, to take into account human variability.

In conclusion, our study has highlighted the potential utility of a leukocyte marker-based screening pipeline aimed at characterizing cancer-specific leukocyte marker changes. The screen facilitates phenotyping of cancer-specific leukocyte populations, the counts of which can be used for ML model training to predict the cancer presence and type within the constraints of the dataset. Furthermore, it has the potential to identify novel cancer-associated leukocyte subsets for further mechanistic investigation. The pipeline can be easily adopted for biomarker detection in clinical samples, which we hypothesize will form the foundation of ML models to aid clinical decision-making in the future.

## Data availability statement

The original contributions presented in the study are included in the article/**Supplementary Material**, further inquiries can be directed to the corresponding author/s.

## Ethics statement

Animals were housed in a specific pathogen-free environment and used under strict adherence to protocols approved by the institutional Animal Experimentation Ethics Committee (AEEC), Australian National University (ANU), under protocol A2020/39.

## Author contributions

BQ contributed to the study design, acquisition, analysis and interpretation of data, drafting of the manuscript, and final manuscript approval. DS contributed to the study design, data acquisition, drafting of the manuscript, and final manuscript approval. MR contributed to data acquisition, and final manuscript approval. IA, DY, KJ, and FS contributed to the study design, drafting of the manuscript, and final manuscript approval. DH contributed to the study analysis. KG, JG, RS, NO, AO and JP contributed to data acquisition and drafting of the manuscript and final manuscript approval.
