# Supplementary material for: Identifying cancer-associated leukocyte profiles using high-resolution flow cytometry screening and machine learning
Source: Front Immunol. 2023 Aug 3;14:1211064. doi: 10.3389/fimmu.2023.1211064 (PMC10435879; doi:10.3389/fimmu.2023.1211064)
Supplement: Supplementary file 2 [file DataSheet_1.docx]

Supplementary Material

**Identifying cancer-associated leukocyte profiles using high-resolution flow cytometry screening and machine learning**

David A Simon Davis^†^, Melissa Ritchie^†^, Dillon Hammill, Jessica Garrett, Robert O Slater, Naomi Otoo, Anna Orlov, Katharine Gosling, Jason Price, Desmond Yip, Kylie Jung, Farhan M Syed, Ines I Atmosukarto and Ben J C Quah

^†^Contributed equally to the work

^*^Corresponding author, [ben.quah@anu.edu.au](mailto:ben.quah@anu.edu.au); +61 (0)2 6125 2439; John Curtin School of Medical Research, Australian National University, 131 Garran Rd, Acton ACT 2601, Australia

**S1 Table: Barcoding vital dye cell-labelling concentrations**

| **Group** | **Sequence** | **CFSE concentration** | **CTV concentration** |
| --- | --- | --- | --- |
| Nil BALB/c | 1 | 74 nM | 0 nM |
| CT26 | 2 | 74 nM | 1500 nM |
| 4T1 | 3 | 74 nM | 20300 nM |
| B16-F10 | 4 | 11 nM | 0 nM |
| MC38 | 5 | 11 nM | 1500 nM |
| AT3-OVA | 6 | 11 nM | 20300 nM |
| 4T1.Br4 | 7 | 0 nM | 0 nM |
| 4T1.2 | 8 | 0 nM | 1500 nM |
| Nil C57BL/6 | 9 | 0 nM | 20300 nM |

**S2 Table: Antibodies**

| **Panel** | **Antigen** | **Clone** | **Fluorochrome** | **Cat. #** | **Isotype**  (r = rat; ah = Armenian hamster) | **Source** | **2 X Stock dilution factor** |
| --- | --- | --- | --- | --- | --- | --- | --- |
| **Backbone** | CD45 | 30-F11 | PerCP-Cy5.5 | 103132 | r-IgG2b, k | Biolegend | 1/50 |
|  | CD90.2 | 53-2.1 | PE-Cy7 | 105326 | r-IgG2b, k | Biolegend | 1/200 |
|  | CD4 | RM4-5 | AF-700 | 100536 | r-IgG2a,k | Biolegend | 1/400 |
|  | CD8a | 53-6.7 | BV650 | 100742 | r-IgG2a,k | Biolegend | 1/50 |
|  | PD-1 | 29F.1A12 | APC | 135210 | r-IgG2a,k | Biolegend | 1/400 |
|  | CD25 | PC61 | APC-F750 | 102054 | r-IgG2a, l | Biolegend | 1/400 |
|  | B220 | RA3-6B2 | AF-700 | 103232 | r-IgG2a,k | Biolegend | 1/50 |
|  | CD11c | N418 | APC | 117310 | ah-IgG | Biolegend | 1/100 |
|  | CD11b | M1/70 | APCFire750 | 101262 | r-IgG2b, k | Biolegend | 1/400 |
|  | Ly-6C | HK1.4 | BV711 | 128037 | r-IgG2c,k | Biolegend | 1/50 |
|  | Ly-6G | 1A8 | BV650 | 127606 | r-IgG2a,k | Biolegend | 1/100 |
|  | F4/80 | BM8 | PE-Cy7 | 123114 | r-IgG2a,k | Biolegend | 1/100 |
|  | I-A/I-E (MHC-II) | M5/114.15.2 | BV605 | 107639 | r-IgG2b, k | Biolegend | 1/50 |
|  | PD-L1 | 10F.9G2 | PE-Dazzle594 | 124324 | r-IgG2b, k | Biolegend | 1/50 |
|  | Siglec-F | E50-2440 | BV786 | 740956 | r-IgG2a,k | BD | 1/50 |
|  | CD49b | DX5 | BUV395 | 740250 | ah-IgG1, k | BD | 1/400 |
|  | TCRb | H57-597 | BV605 | 109241 | ah-IgG | Biolegend | 1/50 |
| **Backbone + screen markers** | CD62L | MEL-14 | BV570 | 104433 | r-IgG2a,k | Biolegend | 1/50 |
|  | CD44 | IM7 | BUV737 | 612799 | r-IgG2b, k | BD | 1/50 |
|  | CD24 | M1/69 | PE | 101808 | r-IgG2b,k | Biolegend | 1/200 |
|  | CD45RB | C363-16A | FITC | 103305 | r-IgG2a,k | Biolegend | 1/100 |
|  | IgD | 11-26c.2a | BV421 | 405725 | r-IgG2a,k | Biolegend | 1/100 |
|  | CD66a | Mab-CC1 | BV650 | 134529 | m-IgG1, k | Biolegend | 1/100 |


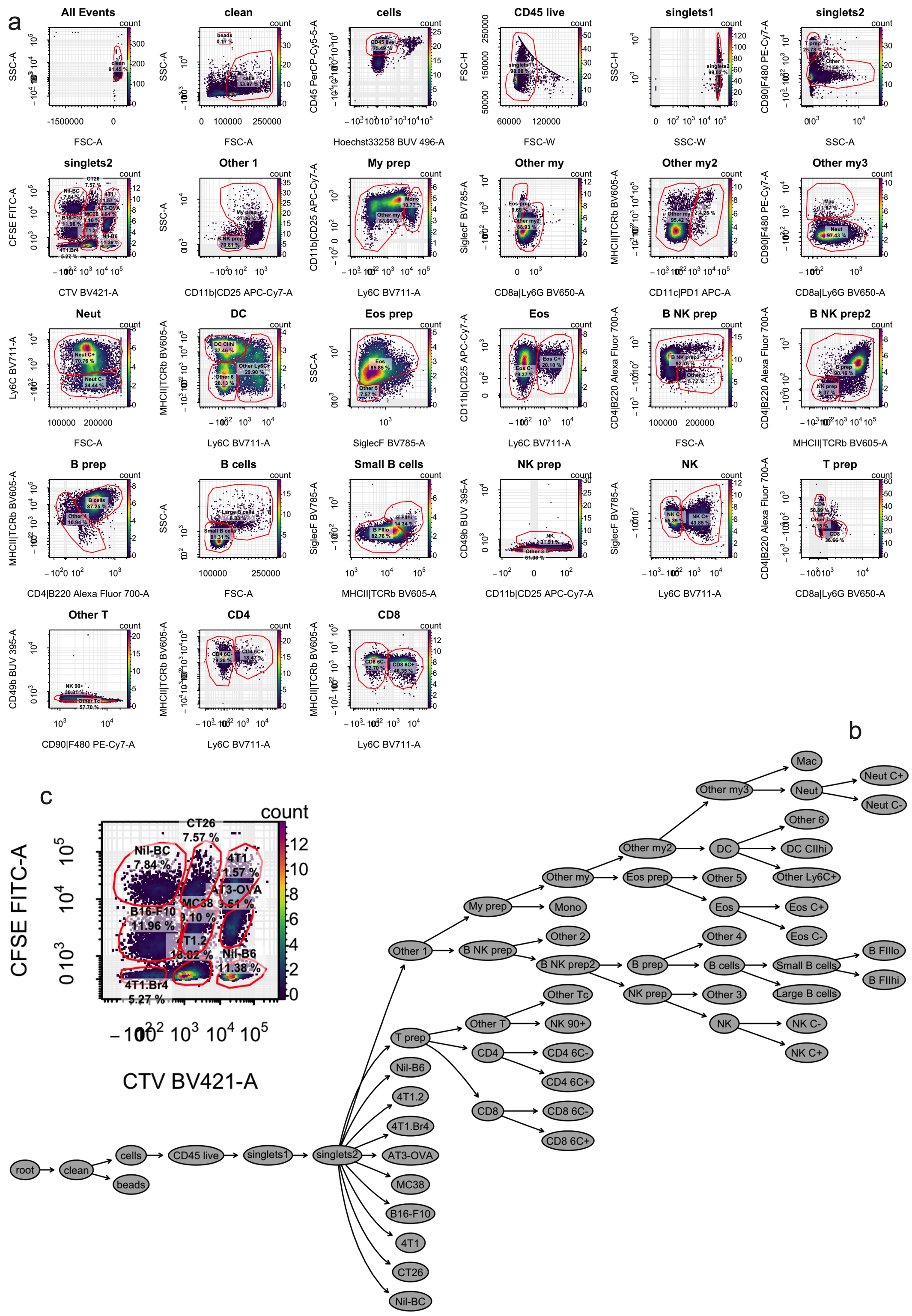


**S1 Figure: Spleen and blood leukocyte manual gating strategy**

Spleen and blood cell samples were analysed by flow cytometry as described in Fig 3. Two dimensional plots depicting the manual gating strategy performed using the R package CytoExploreR to delineate leukocyte subsets (based on the backbone antibody-labelling) and barcoded populations (based on the vital dye labelling) (a). The hierarchy of the gating order is plotted in (b). The population gates starting from and including the “Other 1” and “T prep” gates were also placed on each barcoded population for the screening assay but are not shown due to complexity. A close up of the barcoded populations is shown in (c).


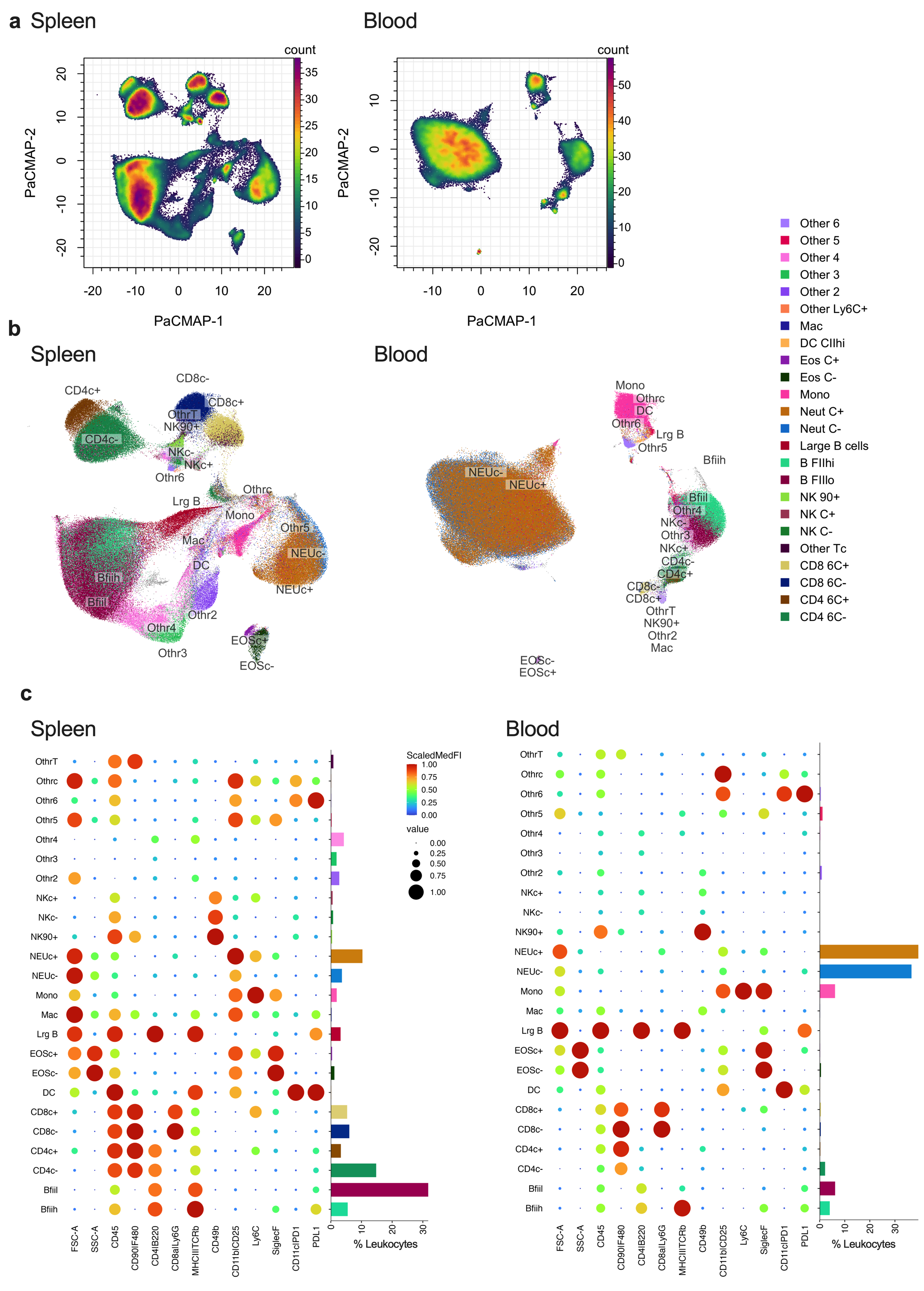


**S2 Figure: Spleen and blood leukocyte backbone marker phenotypes**

Spleen and blood cells were harvested from tumour-bearing and no-tumour controls for use in the screening pipeline (as described in Fig 2). Pooled replicate spleen samples (from the 3 most tumour-burdened animals) were barcoded with the vital dyes CFSE and CTV to generate group-specific discrete fluorescence spectra and all spleen groups pooled. Barcoded pooled spleen cells and associated blood samples (from each group) were labelled with a backbone panel of leukocyte phenotyping fluorescently tagged antibodies and samples analysed by flow cytometry. Live singlet CD45^+^ leukocytes were gated (as described in S1 Fig) and assessed for population segregation using PaCMAP dimensional reduction on pooled groups (a). Manual gating (S1 Fig) was used to delineate leukocyte subsets and these overlayed on PaCMAP plots (b). Marker expression was assessed on all CD45^+^ delineated leukocyte subpopulations and presented as MedFI for each antibody scaled from 0 to 1 (being no to maximum expression) as a heat map dot plot annotated with percent of each population of total leukocytes (c). Results are presented as pooled groups of n=3 per group.

**S3 Table: Backbone panel-delineated leukocyte subsets**

| **Number** | **Terminal population name** | **Long name** | **Marker phenotype** | **Abbreviated names** |
| --- | --- | --- | --- | --- |
| 1 | CD4 6C- | CD4^+^ T cells Ly6C^-^ | CD90^+^\|SSC^Lo^\|CD4^+^\|CD8^-^\|Ly6C^-^ | CD4c- |
| 2 | CD4 6C+ | CD4^+^ T cells Ly6C^+^ | CD90^+^\|SSC^Lo^\|CD4^+^\|CD8^-^\|Ly6C^+^ | CD4c+ |
| 3 | CD8 6C- | CD8^+^ T cells Ly6C^-^ | CD90^+^\|SSC^Lo^\|CD4^-^\|CD8^+^\|Ly6C^-^ | CD8c- |
| 4 | CD8 6C+ | CD8^+^ T cells Ly6C^+^ | CD90^+^\|SSC^Lo^\|CD4^-^\|CD8^+^\|Ly6C^+^ | CD8c+ |
| 5 | Other T | Other T cells | CD90^+^\|SSC^Lo^\|CD4^-^\|CD8^-^\|CD49b^-^ | OthrT |
| 6 | NK C- | Natural Killer cells Ly6C^-^ | CD90^-^\|SSC^Lo^\|CD11b^-^\|B220^-^\|MHC-II^-^\|CD49b^+^\|CD11b^-/+^\|Ly6C^-^ | NKc- |
| 7 | NK C+ | Natural Killer cells Ly6C^+^ | CD90^-^\|SSC^Lo^\|CD11b^-^\|B220^-^\|MHC-II^-^\|CD49b^+^\|CD11b^-/+^\|Ly6C^+^ | NKc+ |
| 8 | NK 90+ | Natural Killer cells CD90^+^ | CD90^+^\|SSC^Lo^\|CD4^-^\|CD8^-^\|CD49b^+^ | NK90+ |
| 9 | B FIILo | B cells MHC-II^Lo^\| SiglecF^-^ | CD90^-^\|SSC^Lo^\|CD11b^-^\|B220^+^\|MHC-II^Lo-Hi^\|FSC^LO^\|SiglecF^-^ | Bfiil |
| 10 | B FIIhi | B cells MHC-II^Hi^\| SiglecF^+^ | CD90^-^\|SSC^Lo^\|CD11b^-^\|B220^+^\|MHC-II^Hi^\|FSC^LO^\|SiglecF^+^ | Bfiih |
| 11 | Large B cells | B cells SSC^Hi^\|FSC^Hi^ | CD90^-^\|SSC^Lo^\|CD11b^-^\|B220^+^\|MHC-II^Lo-Hi^\|FSC^Hi^ | Lrg B |
| 12 | Neut C- | Neutrophils Ly6C- | CD90^-^\|SSC^Lo-Hi^\|CD11b^+^\|Ly6C^-^\|SiglecF^-^\|CD11c^-^\|F4/80^-^\|Ly6G^Lo-Hi^\|Ly6C^-^ | NEUc- |
| 13 | Neut C+ | Neutrophils Ly6C+ | CD90^-^\|SSC^Lo-Hi^\|CD11b^+^\|Ly6C^-^\|SiglecF^-^\|CD11c^-^\|F4/80^-^\|Ly6G^Lo-Hi^\|Ly6C^+^ | NEUc+ |
| 14 | Mono | Monocyte | CD90^-^\|SSC^Lo-Hi^\|CD11b^+^\|Ly6C^+^ | Mono |
| 15 | Eos C- | Eosinophils | CD90^-^\|SSC^Hi^\|  CD11b^+^\|SiglecF^+^\|Ly6C^-^ | EOSc- |
| 16 | Eos C+ | Eosinophils | CD90^-^\|SSC^Hi^\|  CD11b^+^\|SiglecF^+^\|Ly6C^+^ | EOSc+ |
| 17 | DC CIIhi | Dendritic cells MHC-II^Hi^ | CD90^-^\|SSC^Lo-Hi^\|CD11b^+^\|Ly6C^-^\|SiglecF^-^\|CD11c^+^\|MHC-II^Hi^ | DC |
| 18 | Mac | Macrophage | CD90^-^\|SSC^Lo-Hi^\|CD11b^+^\|Ly6C^-^\|SiglecF^-^\|CD11c^-^\|F4/80^+^ | Mac |
| 19 | Other Ly6C+ | Other Ly6C^+^ unknown cells | CD90^-^\|SSC^Lo-Hi^\|CD11b^+^\|Ly6C^+^\|SiglecF^-^\|CD11c^+^\|MHC-II^Lo-Hi^ | Othrc |
| 20 | Other 2 | Other unknown cells 2 | CD90^-^\|SSC^Lo^\|CD11b^-^\|B220^-^\|FSC^Int-Hi^ | Othr2 |
| 21 | Other 3 | Other unknown cells 3 | CD90^-^\|SSC^Lo^\|CD11b^-^\|B220^-^\|MHC-II^-^\|CD49b^-^ | Othr3 |
| 22 | Other 4 | Other unknown cells 4 | CD90^-^\|SSC^Lo^\|CD11b^-^\|B220^Lo-Hi^\|MHC-II^Lo-Hi^\|FSC^LO^\|SiglecF^+^ | Othr4 |
| 23 | Other 5 | Other unknown cells 5 | CD90^-^\|SSC^Lo^\|  CD11b^+^\|SiglecF^+^ | Othr5 |
| 24 | Other 6 | Other unknown cells 6 | CD90^-^\|SSC^Lo-Hi^\|CD11b^+^\|Ly6C^-^\|SiglecF^-^\|CD11c^+^\|MHC-II^Lo^ | Othr6 |


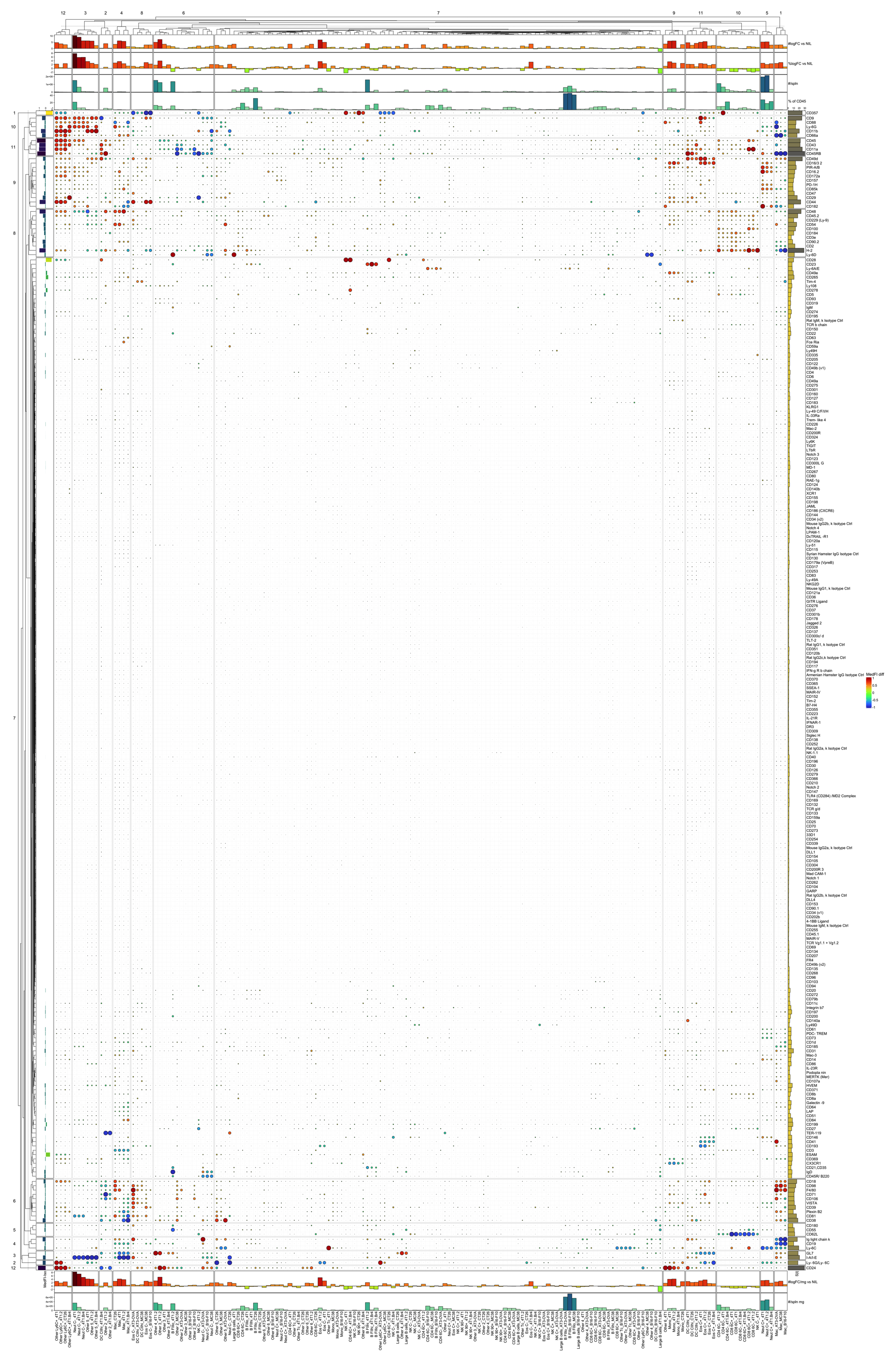


**S3 Figure: LEGENDScreen cell surface marker changes on 24 leukocyte populations across 7 cancer groups**

Barcoded and backbone antibody-labelled spleen samples from tumour-bearing and no-tumour control animals (as described in Fig 3) were equally aliquoted and labelled across all the PE-tagged antibodies in the LEGENDScreen assay and analysed by flow cytometry. After delineating the 7 tumour and 2 no-tumour control groups and the 24 leukocyte populations from each group (as described in S1 Fig), the change in MedFI-PE of each LEGENDScreen antibody across all leukocytes in the tumour samples relative to their background no-tumour controls was calculated and scaled from -1 (being the most extreme decrease in MedFI-PE) to +1 (being the most extreme increase in MedFI-PE), with a value of 0 being identical to background controls. These values were then plotted as a heat map dot plot, with dot size reflecting absolute change in MedFI-PE. Hierarchical clustering using Euclidean distance was used to group the markers or populations and the relationships summarised using dendrograms. The most related clusters were partitioned into 12 groups on each axis. The expression of each marker was also plotted after subtracting the MedFI-PE of a matched isotype control antibody from its average MedFI-PE (left bar plot annotation). The sum of all absolute MedFI-PE changes (S|I|) was also calculated across the groups for each marker (right bar plot annotation). Finally, enumeration of each CD45^+^ leukocyte subpopulation based on either % of total CD45^+^ leukocytes or total cell number (#) was calculated per spleen (top annotations) or per milligram (mg) of spleen (bottom annotations) and included log2 fold changes (logFC) from no-tumour control (Nil) levels. Results are from pooled samples of n=3 per group.


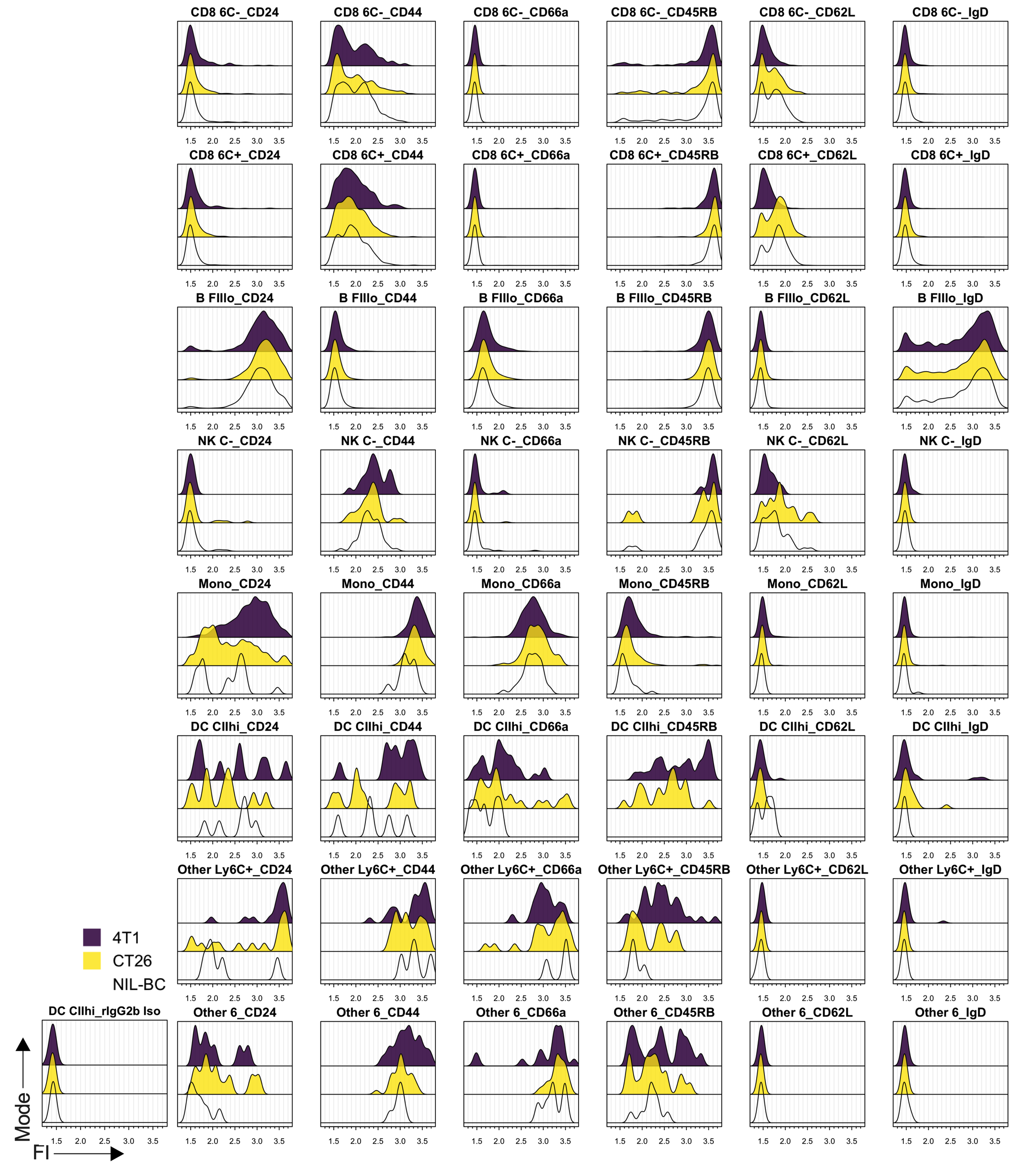


**S4 Figure: LEGENDScreen surface marker changes in leukocytes from CT26, 4T1 and no-tumour control groups**

Live CD45^+^ leukocytes from CT26- (yellow-filled histogram), 4T1- (maroon-filled histogram)-bearing and no-tumour (open histogram) BC control animals (as described in Fig 3) were analysed by the LEGENDScreen pipeline (S2 Fig). Changes in pipeline-identified markers and populations from CT26, 4T1 and no-tumour samples were plotted as overlaid histograms including a representative isotype antibody control (rat IgG2b) on a dendritic cell population (DC CIIhi_rIgG2b iso). Each plot has the name of the leukocyte followed by the marker in its title separated by an underscore. Each histogram represents a sample pooled from n=3.


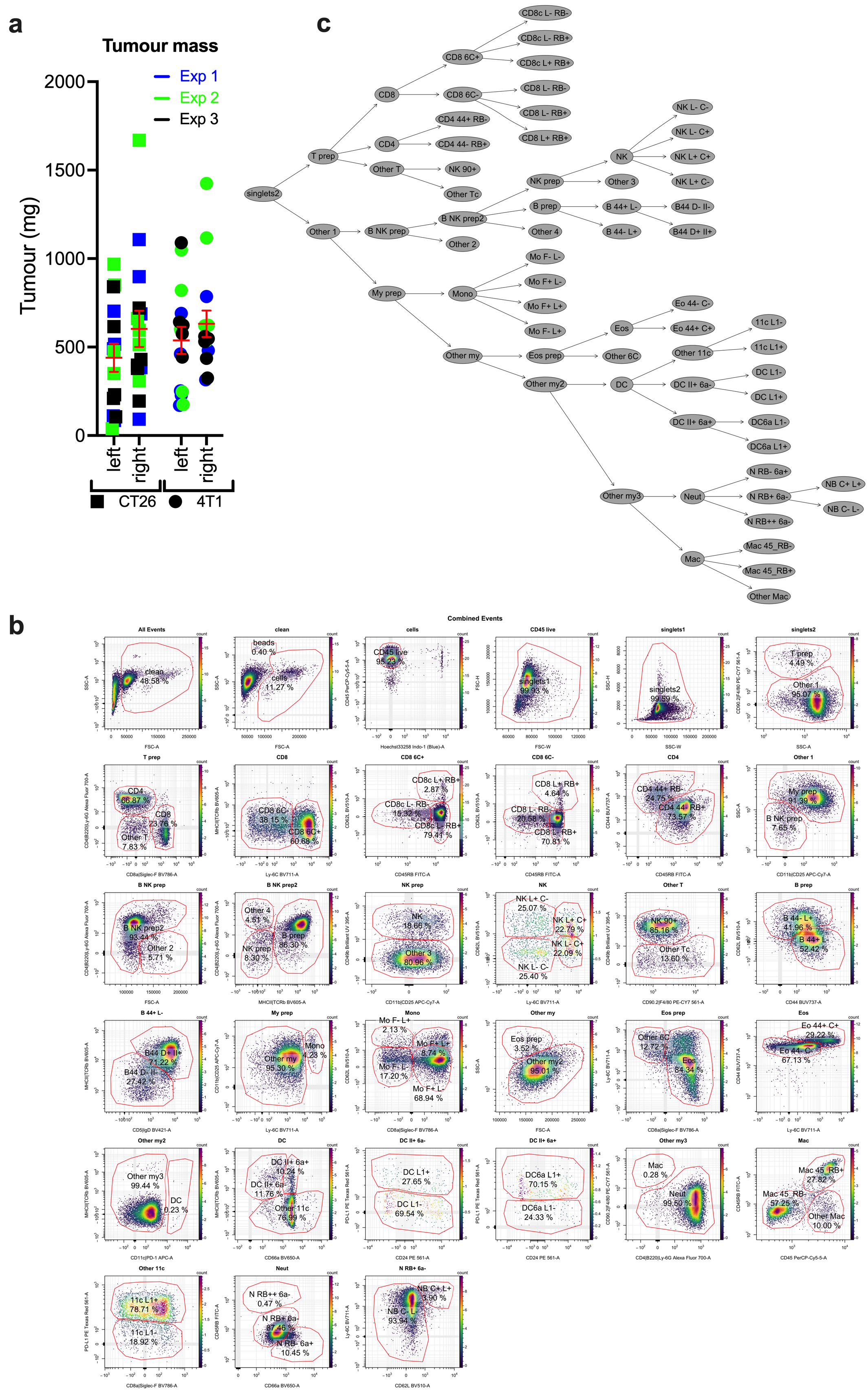


**S5 Figure: Spleen and blood leukocyte phenotyping using pipeline-informed labelling and manual gating**

CT26 and 4T1 tumours were established subcutaneously in the right- (day-0) and left- (day-3) hind flanks of adult female syngeneic (BC) mice. At study end-point (day-21), mice were humanely sacrificed and their tumours extracted and weighed with individual weights and means and standard error of means displayed (red lines) (a). Data are from 3 independent experiments (annotated Exp 1-3), each with 5 replicates per group. Blood cells from animals were also labelled with an expanded panel of antibodies to include pipeline-identified tumour-associated markers of interest in addition to the existing backbone antibodies, and analysed by flow cytometry. Manual gating was performed using the R package CytoExploreR to delineate leukocyte subsets (b), the hierarchy of which is plotted in (c).

**S4 Table: Screen-defined panel delineated leukocyte subsets**

| **Number** | **Terminal population name** | **Long name** | **Marker phenotype** | **Abbreviated names** |
| --- | --- | --- | --- | --- |
| 1 | CD4 44+ RB- | CD4^+^ T cells CD44^Hi^\|CD45RB^Lo^ | CD90^+^\|SSC^Lo^\|CD4^+^\|CD8^-^\|CD45RB^Lo^\|CD44^Hi^ | CD4 44+B- |
| 2 | CD4 44- RB+ | CD4^+^ T cells Ly6C^+^ | CD90^+^\|SSC^Lo^\|CD4^+^\|CD8^-^\|CD45RB^Hi^\|CD44^Lo^ | CD4 44-B+ |
| 3 | CD8 L-RB- | CD8^+^ T cells Ly6C^-^\|CD62L^-^\|CD45RB^-^ | CD90^+^\|SSC^Lo^\|CD4^-^\|CD8^+^\|Ly6C^-^\|CD62L^-^\|CD45RB^-^ | CD8 L-B- |
| 4 | CD8 L-RB+ | CD8^+^ T cells Ly6C^-^\|CD62L^-^\|CD45RB^+^ | CD90^+^\|SSC^Lo^\|CD4^-^\|CD8^+^\|Ly6C^-^\|CD62L^-^\|CD45RB^+^ | CD8 L-B+ |
| 5 | CD8 L+RB+ | CD8^+^ T cells Ly6C^-^\|CD62L^+^\|CD45RB^+^ | CD90^+^\|SSC^Lo^\|CD4^-^\|CD8^+^\|Ly6C^-^\|CD62L^+^\|CD45RB^+^ | CD8 L+B+ |
| 6 | CD8c L-RB- | CD8^+^ T cells Ly6C^+^\|CD62L^-^\|CD45RB^-^ | CD90^+^\|SSC^Lo^\|CD4^-^\|CD8^+^\|Ly6C^+^\|CD62L^-^\|CD45RB^-^ | CD8c L-B- |
| 7 | CD8c L-RB+ | CD8^+^ T cells Ly6C^+^\|CD62L^-^\|CD45RB^+^ | CD90^+^\|SSC^Lo^\|CD4^-^\|CD8^+^\|Ly6C^+^\|CD62L^-^\|CD45RB^+^ | CD8c L-B+ |
| 8 | CD8c L+RB+ | CD8^+^ T cells Ly6C^+^\|CD62L^+^\|CD45RB^+^ | CD90^+^\|SSC^Lo^\|CD4^-^\|CD8^+^\|Ly6C^+^\|CD62L^+^\|CD45RB^+^ | CD8c L+B+ |
| 9 | Other Tc | Other T cells | CD90^+^\|SSC^Lo^\|CD4^-^\|CD8^-^\|CD49b^-^ | Othr T |
| 10 | NK L-C- | Natural Killer cells \|CD62L^-^\|Ly6C^-^ | CD90^-^\|SSC^Lo^\|CD11b^-^\|B220^-^\|MHC-II^-^\|CD49b^+^\|CD11b^-/+^\|CD62L^-^\|Ly6C^-^ | NK L-C- |
| 11 | NK L-C+ | Natural Killer cells \|CD62L^-^\|Ly6C^+^ | CD90^-^\|SSC^Lo^\|CD11b^-^\|B220^-^\|MHC-II^-^\|CD49b^+^\|CD11b^-/+^\|CD62L^-^\|Ly6C^+^ | NK L-C+ |
| 12 | NK L+C+ | Natural Killer cells \|CD62L^+^\|Ly6C^+^ | CD90^-^\|SSC^Lo^\|CD11b^-^\|B220^-^\|MHC-II^-^\|CD49b^+^\|CD11b^-/+^\|CD62L^+^\|Ly6C^+^ | NK L+C+ |
| 13 | NK L+C- | Natural Killer cells \|CD62L^+^\|Ly6C^-^ | CD90^-^\|SSC^Lo^\|CD11b^-^\|B220^-^\|MHC-II^-^\|CD49b^+^\|CD11b^-/+^\|CD62L^+^\|Ly6C^-^ | NK L+C- |
| 14 | NK 90+ | Natural Killer cells CD90^+^ | CD90^+^\|SSC^Lo^\|CD4^-^\|CD8^-^\|CD49b^+^ | NK 90+ |
| 15 | B44-L+ | B cells CD44^-^\|CD62L^+^ | CD90^-^\|SSC^Lo^\|CD11b^-^\|B220^+^\|MHC-II^Lo-Hi^ \|FSC^LO^\|CD44^-^\|CD62L^+^ | B44-L+ |
| 16 | B44+D- II- | B cells CD44^+^\| IgD^-^\|MHC-II^Lo^ | CD90^-^\|SSC^Lo^\|CD11b^-^\|B220^+^\|MHC-II^Lo-Hi^ \|FSC^LO^\|CD44^-^\|CD62L^+^\|IgD^-^\|MHC-II^Lo^ | B44 D-II- |
| 17 | B44+D+ II+ | B cells CD44^+^\|IgD^+^\|MHC-II^Hi^ | CD90^-^\|SSC^Lo^\|CD11b^-^\|B220^+^\|MHC-II^Lo-Hi^ \|FSC^LO^\|CD44^-^\|CD62L^+^\|IgD^+^\|MHC-II^Hi^ | B44 D+II+ |
| 18 | N RB- 6a+ | Neutrophils CD45RB^-^\|CD66a^+^ | CD90^-^\|SSC^Lo-Hi^\|CD11b^+^\|Ly6C^-^\|SiglecF^-^\|CD11c^-^\|F4/80^-^\|Ly6G^Lo-Hi^\|CD45RB^-^\|CD66a^+^ | N B-6a+ |
| 19 | NB C- L- | Neutrophils CD45RB^+^\|CD66a^Int^\|Ly6C^Int^\|CD62L^-^ | CD90^-^\|SSC^Lo-Hi^\|CD11b^+^\|Ly6C^-^\|SiglecF^-^\|CD11c^-^\|F4/80^-^\|Ly6G^Lo-Hi^\|CD45RB^+^\|CD66a^Int^\|Ly6C^Int^\|CD62L^-^ | NB CL- |
| 20 | NB C+ L+ | Neutrophils CD45RB^+^\|CD66a^Int^\|Ly6C^-^\|CD62L^-^ | CD90^-^\|SSC^Lo-Hi^\|CD11b^+^\|Ly6C^-^\|SiglecF^-^\|CD11c^-^\|F4/80^-^\|Ly6G^Lo-Hi^\|CD45RB^+^\|CD66a^Int^\|Ly6C^+^\|CD62L^+^ | NB CL+ |
| 21 | N RB++ 6a+- | Neutrophils CD45RB^Hi^\|CD66a^-^ | CD90^-^\|SSC^Lo-Hi^\|CD11b^+^\|Ly6C^-^\|SiglecF^-^\|CD11c^-^\|F4/80^-^\|Ly6G^Lo-Hi^\|CD45RB^Hi^\|CD66a^-^ | N RB++6a+- |
| 22 | Mo F- L- | Monocyte SiglecF^-^\|CD62L^-^ | CD90^-^\|SSC^Lo-Hi^\|CD11b^+^\|Ly6C^+^\|SiglecF^-^\|CD62L^-^ | Mo F-L- |
| 23 | Mo F+ L- | Monocyte SiglecF^+^\|CD62L^-^ | CD90^-^\|SSC^Lo-Hi^\|CD11b^+^\|Ly6C^+^\|SiglecF^+^\|CD62L^-^ | Mo FL- |
| 24 | Mo F+ L+ | Monocyte SiglecF^+^\|CD62L^+^ | CD90^-^\|SSC^Lo-Hi^\|CD11b^+^\|Ly6C^+^\|SiglecF^+^\|CD62L^+^ | Mo F+ L+ |
| 25 | Mo F- L+ | Monocyte SiglecF^-^\|CD62L^+^ | CD90^-^\|SSC^Lo-Hi^\|CD11b^+^\|Ly6C^+^\|SiglecF^-^\|CD62L^+^ | Mo F-L+ |
| 26 | Eo 44- C- | Eosinophils CD44^-^\|Ly6C^-^ | CD90^-^\|SSC^Hi^\|  CD11b^+^\|SiglecF^+^\|CD44^-^\|Ly6C^-^ | Eo 44-C- |
| 27 | Eo 44+ C+ | Eosinophils CD44^+^\|Ly6C^+^ | CD90^-^\|SSC^Hi^\|  CD11b^+^\|SiglecF^+^\|CD44^+^\|Ly6C^+^ | Eo 44+C+ |
| 28 | DC L1- | Dendritic cells CD66a^-^\|PD-L1^-^ | CD90^-^\|SSC^Lo-Hi^\|CD11b^+^\|Ly6C^-^\|SiglecF^-^\|CD11c^+^\|MHC-II^Hi^\|CD66a^-^\|PD-L1^-^ | DC L1- |
| 29 | DC L1+ | Dendritic cells CD66a^-^\|PD-L1^+^ | CD90^-^\|SSC^Lo-Hi^\|CD11b^+^\|Ly6C^-^\|SiglecF^-^\|CD11c^+^\|MHC-II^Hi^\|CD66a^-^\|PD-L1^+^ | DC L1+ |
| 30 | DC6a L1- | Dendritic cells CD66a^+^\|PD-L1^-^ | CD90^-^\|SSC^Lo-Hi^\|CD11b^+^\|Ly6C^-^\|SiglecF^-^\|CD11c^+^\|MHC-II^Hi^\|CD66a^+^\|PD-L1^-^ | DC6a L1- |
| 31 | DC6a L1+ | Dendritic cells CD66a^+^\|PD-L1^+^ | CD90^-^\|SSC^Lo-Hi^\|CD11b^+^\|Ly6C^-^\|SiglecF^-^\|CD11c^+^\|MHC-II^Hi^\|CD66a^+^\|PD-L1^+^ | DC6a L1+ |
| 32 | Mac 45_RB- | Macrophage CD45^-^\|CD45RB^-^ | CD90^-^\|SSC^Lo-Hi^\|CD11b^+^\|Ly6C^-^\|SiglecF^-^\|CD11c^-^\|F4/80^+^\|CD45^Lo^\|CD45RB^-^ | Mac 45_B- |
| 33 | Mac 45_RB+ | Macrophage CD45^+^\|CD45RB^+^ | CD90^-^\|SSC^Lo-Hi^\|CD11b^+^\|Ly6C^-^\|SiglecF^-^\|CD11c^-^\|F4/80^+^\|CD45^Hi^\|CD45RB^+^ | Mac 45_B+ |
| 34 | Other Mac | Macrophage CD45^+^\|CD45RB^-^ | CD90^-^\|SSC^Lo-Hi^\|CD11b^+^\|Ly6C^-^\|SiglecF^-^\|CD11c^-^\|F4/80^+^\|CD45^Hi^\|CD45RB^-^ | Othr Mac |
| 35 | 11c L1- | Myeloid cells CD11c^+^ MHC-II^-^ CD66a^+^\|PD-L1^-^ | CD90^-^\|SSC^Lo-Hi^\|CD11b^+^\|Ly6C^-^\|SiglecF^-^\|CD11c^+^\|MHC-II^-^\|CD66a^+^\|PD-L1^-^ | 11c L1- |
| 36 | 11c L1+ | Myeloid cells CD11c^+^ MHC-II^-^ CD66a^+^\|PD-L1^+^ | CD90^-^\|SSC^Lo-Hi^\|CD11b^+^\|Ly6C^-^\|SiglecF^-^\|CD11c^+^\|MHC-II^-^\|CD66a^+^\|PD-L1^+^ | 11c L1+ |
| 37 | Other Ly6C | Other Ly6C^+^ unknown cells | CD90^-^\|SSC^Hi^\|CD11b^+^\|Ly6C^Int^\|SiglecF | Othr 6C |
| 38 | Other 3 | Other unknown cells 3 | CD90^-^\|SSC^Lo^\|CD11b^-^\|B220^-^\|MHC-II^-^\|CD49b^-^ | Othr 1 |
| 39 | Other 4 | Other unknown cells 4 | CD90^-^\|SSC^Lo^\|CD11b^-^\|B220^Hi^\|MHC-II^Lo^ | Othr 2 |

**
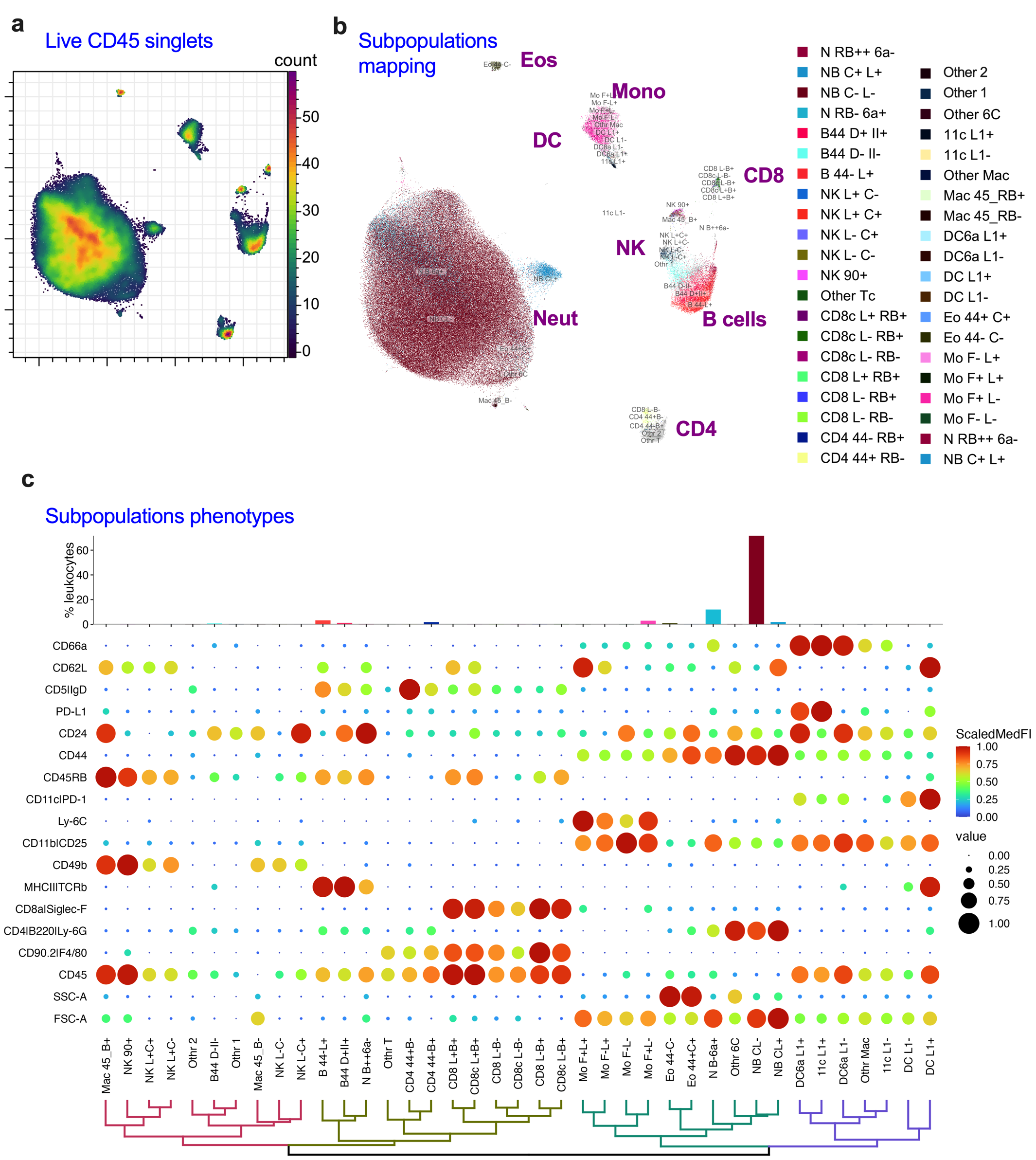
**

**S6 Figure: Blood leukocyte phenotypes using an expanded pipeline-informed antibody panel**

Blood leukocyte populations from CT26- and 4T1-bearing mice and no-tumour controls were identified as described in S4 Fig. Live singlet CD45^+^ leukocytes were gated (S4 Fig) and assessed for population segregation using PaCMAP dimensional reduction (a). Manual gating (S4 Fig) was used to delineate leukocyte subsets and these overlaid on PaCMAP plots (b). Marker expression was assessed on all delineated leukocytes (grouped by hierarchical clustering as presented by the dendrogram) and presented as MedFI for each antibody scaled from 0 to 1 as a heat map dot plot annotated with percent of each population of total leukocytes (c). Results are presented from pooled groups.
